# Mathematical and Computational Modeling of Poroelastic Cell Scaffolds Used in the Design of an Implantable Bioartificial Pancreas

**DOI:** 10.3390/fluids7070222

**Published:** 2022-07-01

**Authors:** Yifan Wang, Sunčica Čanić, Martina Bukač, Charles Blaha, Shuvo Roy

**Affiliations:** 1 Department of Mathematics, University of California, Irvine, CA 92697, USA; 2 Department of Mathematics, University of California, Berkeley, CA 94720, USA; 3 Department of Applied and Computational Mathematics and Statistics, University of Notre Dame, South Bend, IN 46556, USA; 4 Medical Center, University of California, San Francisco, CA 94143, USA; 5 Department of Bioengineering and Therapeutic Sciences, University of California, San Francisco, CA 94143, USA

**Keywords:** bioartificial pancreas, mathematical modeling, fluid-poroelastic structure interaction

## Abstract

We present a multi-scale mathematical model and a novel numerical solver to study blood plasma flow and oxygen concentration in a prototype model of an implantable Bioartificial Pancreas (iBAP) that operates under arteriovenous pressure differential without the need for immunosuppressive therapy. The iBAP design consists of a poroelastic cell scaffold containing the healthy transplanted cells, encapsulated between two semi-permeable nano-pore size membranes to prevent the patient’s own immune cells from attacking the transplant. The device is connected to the patient’s vascular system via an anastomosis graft bringing oxygen and nutrients to the transplanted cells of which oxygen is the limiting factor for long-term viability. Mathematically, we propose a (nolinear) fluid–poroelastic structure interaction model to describe the flow of blood plasma through the scaffold containing the cells, and a set of (nonlinear) advection–reaction–diffusion equations defined on moving domains to study oxygen supply to the cells. These macro-scale models are solved using finite element method based solvers. One of the novelties of this work is the design of a novel second-order accurate fluid–poroelastic structure interaction solver, for which we prove that it is unconditionally stable. At the micro/nano-scale, Smoothed Particle Hydrodynamics (SPH) simulations are used to capture the micro/nano-structure (architecture) of cell scaffolds and obtain macro-scale parameters, such as hydraulic conductivity/permeability, from the micro-scale scaffold-specific architecture. To avoid expensive micro-scale simulations based on SPH simulations for every new scaffold architecture, we use Encoder–Decoder Convolution Neural Networks. Based on our numerical simulations, we propose improvements in the current prototype design. For example, we show that highly elastic scaffolds have a higher capacity for oxygen transfer, which is an important finding considering that scaffold elasticity can be controlled during their fabrication, and that elastic scaffolds improve cell viability. The mathematical and computational approaches developed in this work provide a benchmark tool for computational analysis of not only iBAP, but also, more generally, of cell encapsulation strategies used in the design of devices for cell therapy and bio-artificial organs.

## 1. Introduction

We present a mathematical model and a numerical solver to study a design of an implantable bioartificial pancreas (iBAP) that operates without the need for immunosuppressive drugs. The main purpose of a bioartificial pancreas is to treat Type 1 Diabetes (T1D), which is an autoimmune disease that affects over 1.6 million people in the United States. The current standard of care is glucose monitoring coupled with exogenous insulin administration via injections or pump. Less common interventions, such as transplantation of islets (spheroid-like cell aggregates that contain endocrine cells of the pancreas) or pancreas transplantation, are reserved for those patients for whom insulin therapy does not allow adequate metabolic control and who experience severe hypoglycemic events. The main obstacles for islet transplantation are poor graft function within a few years post transplantation, negative side effects of lifelong immunosuppression, and pancreas donor shortage. A bioartificial pancreas promises to expand islet cell therapy to substantially more T1D patients because of its immunoprotective cell encapsulation design, and because the types of cells that can be used in its design include not only the pancreatic islets but also those derived from human pluripotent stem cells. The human pluripotent stem cells can be used to create mature β-cells found in pancreatic islets, which are responsible for insulin production [1]. This promises solution to the shortage of donor organs as a source of pancreatic islets, while cell encapsulation eliminates the need for immunosuppressants.

A prototype of the bioartificial pancreas, under development in Dr. Roy’s Lab [2–5], consists of a biocompatible hydrogel scaffold containing the transplanted cells, encapsulated between two semi-permeable nanopore silicon membranes. See Figure 1. The silicon membranes are specifically designed for immunoprotection of the encapsulated islets, while enabling high oxygen delivery and high mass transfer rates of glucose and insulin. They protect the transplant from being attacked by the patients immune system (antibodies and cytokines), while allowing passage of oxygen and nutrients necessary for long-term viability of the organ. The membranes are surface-modified with polyethylene glycol (PEG) to inhibit protein adsorption, fouling, and thrombosis [2].

The encapsulated cell chamber is connected to anastomosis grafts, which connect the device to the patient’s vascular system. See Figure 2. One anastomosis graft brings oxygen and nutrients rich blood to the cell chamber, while the other transports away the insulin produced by the cells. The anastomosis grafts are connected to an artery on one side, and a vein on the other, thereby generating sufficient pressure gradient for significant flow within the anastomosis graft. See the sketch in Figure 2, which shows the device implanted in a patient’s arm and connected to the radial artery on one end, and a vein on the other.

One of the key challenges in bioartificial pancreas design is sufficient oxygen supply to the transplanted cells within the hydrogel scaffold.

To increase oxygen concentration in the cell chamber (poroelastic hydrogel), ultrafiltrate channels are drilled within the hydrogel for advection enhanced oxygen supply. See Figure 3.

In this manuscript we study the design and performance of a second generation implantable Bioartificial Pancreas prototype. A sketch of the prototype device is shown in Figure 4.

This figure shows the device with the inflow-outflow channel, and four islet chambers, two on either side of the channel. The inlet to the channel is connected via an anastomosis graft to an artery, and the outlet to a vein. See Figure 5, which shows a prototype implantable Bioartificial Pancreas (iBAP) implanted into a porcine model. A pressure drop between an artery and a vein drives the blood flow through the channel. Each of the four islet chambers is connected to the blood-supplying channel through a number of semipermeable silicon membranes. The nutrients rich blood flow gets filtered through the membranes and the filtered blood plasma further flows through a gasket and into a hydrogel containing the transplanted cells. As the nutrients rich blood plasma passes through the hydrogel, it feeds the pancreatic islets, and it picks up the produced insulin, which is then carried away from the hydrogel through a gasket with an attached outlet (ultrafiltrate outlet; see Figure 4). The four ultrafiltrate outlets (each associated with one islet chamber) are connected via a catheter to a vein, which receives insulin rich blood plasma.

Islet chamber details as used in the design of the computational domain, are presented in the sketch shown in Figure 6. The blood channel supplying oxygen and nutrients rich blood to the islet chamber is not shown in Figure 6. The four membranes are shown in Figure 6 at the bottom of the sketch with the “inlet” arrows pointing in the flow direction. Two (rectangular) membranes are in the front, and two in the back. The thin gasket region containing the oxygen and nutrients rich blood plasma is adjacent to the islet chamber (poroelastic medium containing the cells), shown in orange color. Figure 6 also shows ultrafiltrate channels in orange color, distributed throughout the islet chamber. The ultrafiltrate flow that passes through the cell chamber enters the top gasket region which collects the insulin rich blood plasma. The insulin rich blood plasma exits the gasket through an ultrafiltrate outlet and enters the “returning” part of the anastomosis graft (not shown in this sketch) connected to the patient’s vein.

The main goal of this manuscript is to design a multi-scale mathematical model and a computational software to study fluid flow (blood plasma) and oxygen concentration within the bioartificial pancreas, which can be used to study the performance of the current design and suggest improvements in terms of increased oxygen supply to the transplanted cells. The key mathematical goals are: (1) To capture the interaction between blood plasma (a Newtonian viscous, incompressible fluid) and a poroelastic medium (cell scaffold), which is a hydrogel in which the permeability properties depend on the fluid content [7], thereby giving rise to a **nonlinear** mathematical model, (2) To capture oxygen concentration in the cell chamber and the gasket. In both of these goals it is important to capture the micro-structure of the hydrogel, which calls for multi-scale modeling.

As we shall see below, elasticity of cell scaffolds is important in studying filtrate flow and oxygen concentration in cell scaffolds. This is especially important since elasticity can be controlled in the fabrication of agarose hydrogel scaffolds, fabricated using the approaches presented in [1]. Mathematically, devising a higher-order accurate fluid–structure interaction computational solver capturing poroelasticity of the scaffolds in which permeability depends on the fluid content is highly nontrivial. In this manuscript we design such a solver and prove, using rigorous stability estimates, that the resulting numerical solver for a corresponding linearized problem is unconditionally stable. This is one of the mathematical novelties of this work.

More specifically, in this manuscript we present a multi-scale model consisting of the following. At the **macro scale**, we consider the following partial differential Equations (PDE) models:

1. A fluid–structure interaction (FSI) model describing the interaction between the blood plasma modeled by the Navier–Stokes or time-dependent Stokes equations for an incompressible, viscous fluid, and a poroelastic hydrogel containing the cells, modeled by the nonlinear Biot Equations (see Section 2.1). The nonlinearity in the Biot equations comes from the dependence of the hydrogel’s permeability on fluid content/porosity [7];
2. Two advection–reaction–diffusion models describing oxygen concentration within the poroelastic hydrogel containing the cells, and oxygen concentration within the gasket containing blood plasma (see Section 2.2). The two models are coupled to the FSI model above through the fluid advection velocity, and through the information about the domain motion. Additionally, the two advection–reaction–diffusion models are coupled among themselves across the interface Γ(t) separating the gasket region from the poroelastic hydrogel scaffold. The coupling conditions describe oxygen transfer from the gasket region to the poroelastic scaffold.

The FSI model and the advection–reaction–diffusion models are solved using Finite-Element Method based numerical solvers. Of particular interest is a second-order accurate fluid–poroelastic structure interaction solver that we introduce in this manuscript, and for which we prove that it is unconditionally stable for the FSI problem with linearized interface motion (fixed fluid domain). This is presented in Section 3.1.

At the **micro scale**, a particle-based Smoothed Particle Hydrodynamics (SPH) model is used to simulate the micro-scale 3D poroelastic structures of hydrogels and calculate the local hydraulic conductivity for every “control” sub-volume of the 3D poroelastic hydrogel. This information is then used to **couple the micro and macro-scale** simulations by obtaining the 3D macro-scale permeability tensor κ from the local fluid content obtained from the micro-scale SPH simulations. See Section 2.1.

To avoid the time-consuming and computationally expensive SPH simulations for every new hydrogel structure, we use Encoder–Decoder Convolution Neural Networks, trained on a set of our synthetic data (calculated off-line), to obtain κ for new hydrogel structures which are either generated synthetically, or for the actual hydrogel structures that can be imaged using high precision laser scanning confocal microscopy.

Micro- and macro-scale coupling is also used at the **inlet**, where we impose macroscale inlet flow data, see (20) below, obtained from the pressure data in the anastomosis graft coupled with the **micro-scale** membrane parameters such as the membrane thickness, pore size, etc. using a Darcy-type relationship (19) derived from experiments with the actual silicon nanopore size membranes considered in this study [2].

Finally, we use our mathematical models and numerical solvers to simulate filtration flow and oxygen concentration for a prototype Bioartificial Pancreas, shown in Figures 4 and 6. The results of our numerical simulation are presented in Section 4. Based on the simulations we propose improvements in the design of the implantable Bioartificial Pancreas, which are discussed in Sections 4 and 5.

Conclusions are presented in Section 5.

To the best of our knowledge, this is the first multi-scale, 3D mathematical and computational model of a bioartificial pancreas, which captures plasma flow interacting with a cell-seeded scaffold and oxygen concentration within the pancreas. Previous models usually address “subsets” of the bioartificial pancreas design, such as, e.g., oxygen concentration and insulin secretion by pancreatic islets. In particular, we mention here an excellent study by Buchwald [8] that informed our own work in terms of advection–reaction–diffusion models for oxygen concentration, where an advection–reaction–diffusion model and the parameters were provided. These parameters and a simplified oxygen concentration and consumption computational model was recently utilized in a study of a simplified bioartificial pancreas without membrane encapsulation, consisting of an acellular tubular graft “lined” with pancreatic islets coated on the outer surface using a hydrogel carrier [9]. In a similar set-up, the work in [10] investigated an in vitro cylindrical perfusion system to study oxygen effects on islet-like clusters immobilized in alginate hydrogel. No computational model for the set up was presented in this work.

The next step of the model development for bioartifical pancreas is modeling glucose-stimulated insulin secretion by the β-cells of pancreatic islets. In [11,12] Buchwald et al. developed a mathematical model and a Finite Element Method solver to study insulin secretion in avascular pancreatic islets that can be used to calculate insulin secretion for arbitrary geometries of cultured, perifused, transplanted, or encapsulated islets in response to various glucose profiles. The model was further used in [13] to study dynamic perifusion with isolated human islets.

However, none of the previous models considered the complexity of an artificial pancreas design such as the one presented in this paper. One of the main novelties of this paper is the fluid–structure interaction model that simulates blood plasma filtration through a poroelastic cell scaffold, which is then coupled to an oxygen concentration model, and in a further study, to an insulin secretion model. This has not been carried out before in the context of a bioartificial pancreas design. Including poroelasticity, as we do in this work, is crucial for manufacturing cell scaffolds with “optimal” elasticity properties for long term cell viability. More information about fluid–poroelastic structure interaction can be found in [14–22]. None of those models, however, were studied in the context of a bioartificial pancreas design.

We remark that the mathematical and computational approaches presented in this work can be used not only for the design of a bioartificial pancreas presented here, but also, more generally, for the analysis of encapsulation strategies used in the design of devices for cell therapy and bio-artificial organs [23].

## 2. The Maco-Scale Mathematical Models

We present two sets of models: one describing the flow of blood plasma in the gasket and in the poroelastic scaffold, presented in Section 2.1, and the other describing concentration of oxygen in the gasket and in the poroelastic scaffold, presented in Section 2.2 below. The equations for oxygen concentration are coupled to the fluid flow model via advection velocity and the fluid domain motion, namely, the equations for oxygen concentration in the gasket and in the hydrogel are solved on *moving domains*. As we show below, including hydrogel elasticity and simulations on moving domains is significant for the analysis of oxygen concentration in highly elastic hydrogels.

We start by presenting details of the fluid–structure interaction (FSI) model describing the blood plasma flow through the gasket and poroelastic hydrogel.

### 2.1. A Fluid-Structure Interaction Model for Blood Plasma and Poroelastic Scaffold

As mentioned earlier, blood plasma enters the islet chamber through four nano-pore size membranes. See Figure 6. The membranes are located at the inlet of the gasket region containing the blood plasma. We will use $\Omega_{f}(t)$ to denote the fluid filled gasket region, and $\Gamma_{\mathrm{in}}$ to denote the part of the boundary of $\Omega_{f}(t)$ corresponding to the inlet. The dependence on t of $\Omega_{f}(t)$ denotes the fact that the fluid domain changes as a function of time due to the interaction between the fluid flow and the poroelastic scaffold sitting on top of $\Omega_{f}(t)$. The reference (fixed) configuration of the fluid domain will be denoted by ${\overset{ˆ}{\Omega }}_{f}$. The poroelastic scaffold region/domain, which is adjacent to $\Omega_{f}(t)$ will be denoted by $\Omega_{p}(t)$, and its reference configuration by ${\overset{ˆ}{\Omega }}_{p}$. Even though the poroelastic scaffold region is moving, the equations are typically written on a fixed, reference domain ${\overset{ˆ}{\Omega }}_{p}$. We denote by Γ(t) the moving interface separating the gasket region from the poroelastic scaffold region, with its reference configuration denoted by $\overset{ˆ}{\Gamma }$.

In the case when poroelastic scaffold contains ultrafiltrate channels, the fluid domain is extended to the channels as well. In this case the boundary between the channels and the poroelastic scaffold is also assumed elastic, and is a part of the fluid domain boundary Γ(t). See Figure 7 below.

#### The fluid model.

To model the flow of blood plasma in the gaskets (and in the ultrafiltrate channels) as shown in Figure 6, we use the Navier–Stokes equations for an incompressible, viscous, Newtonian fluid. Since the Reynolds number in the gasket flow is relatively small, the time-dependent Stokes equations are also adequate. The Navier–Stokes equations defined on $\Omega_{f}(t)$ are given by:

(1) $$\left.\begin{array}{l} \rho_{f}\left(\frac{\partial u_{f}}{\partial t}+\left(u_{f}\cdot \nabla \right)u_{f}\right)=\nabla \cdot \sigma_{f}\left(u_{f},p_{f}\right)+F_{f},\\ \nabla \cdot u_{f}=0, \end{array}\right\}\;\text{in}\;\Omega_{f}(t)\times (0,T),$$

where $u_{f}$ is the fluid velocity, $\sigma_{f}=-p_{f}I+2\mu_{f}D\left(u_{f}\right)$ is the Cauchy stress tensor modeling Newtonian fluid, $p_{f}$ stands for the fluid pressure, $D\left(u_{f}\right)=\left(\nabla u_{f}+{\left(\nabla u_{f}\right)}^{T}\right)/2$ stands for the symmetrized velocity gradient, $\mu_{f}$ is the fluid viscosity, $\rho_{f}$ is the fluid density, and $F_{f}$ denotes the external force term. To close the problem, initial and boundary conditions will be specified in Section 2.1.

#### The poroelastic structure model (cell scaffold).

To model the poroelastic cell scaffold, i.e., the poroelastic structure, we use Biot’s equations of poroelasticity, given by

(2) $$\left.\begin{array}{l} \rho_{p}\frac{\partial^{2}\eta }{\partial t^{2}}=\nabla \cdot \sigma_{p}\left(\eta ,p_{p}\right)+F^{s},\\ \frac{\partial }{\partial t}\left(c_{0}p_{p}+\alpha \nabla \cdot \eta \right)=\nabla \cdot \left(\kappa \nabla p_{p}\right)+F_{p},\\ u_{p}=-\kappa \nabla p_{p}, \end{array}\right\}\;\text{in}\;{\overset{ˆ}{\Omega }}_{p}\times (0,T).$$

The model is given in terms of displacement η of the poroelastic matrix from its reference configuration ${\overset{ˆ}{\Omega }}_{p}$, and the fluid pore pressure $p_{p}$ in the Lagrangian framework, with filtration velocity, $u_{p}$, given in terms of the fluid pore pressure gradient via Darcy's law (third equation in (2)). We note that $u_{p}$ is the *relative* filtration velocity with respect to the poroelastic scaffold motion. The density of the solid material is denoted by $\rho_{p},$ κ is the hydraulic conductivity tensor, and $F^{s}$ and $F_{p}$ are external force and source term, respectively. Coefficient $c_{0}$ is the storage coefficient, α is the Biot-Willis parameter accounting for the coupling strength between the fluid and the solid, and $\sigma_{p}$ is the stress tensor of the poroelastic medium, which is given by $\sigma_{p}=\sigma_{E}-\alpha p_{p}I$, where $\sigma_{E}$ denotes the elasticity stress tensor and I is the identity matrix. To close the system, a constitutive law for $\sigma_{E}(\eta )$ describing the elastic material properties needs to be specified. Here, we assume the linear Saint Venant-Kichhoff material, given by $\sigma_{E}=2\mu_{S}D(\eta )+\lambda_{S}\nabla \cdot \eta I$, where $\mu_{S}$ and $\lambda_{S}$ are Lamé constants. The corresponding initial and boundary conditions will be specified in Section 2.1.

Biot equations have been used to model hydrogel scaffolds by many authors, see, e.g., [7,24,25]. In particular, it was noted that hydrogel’s permeability depends on the fluid content, which is defined as an increment in the volumetric fraction of the fluid component Φ with respect to its reference value $\Phi_{0}$:

(3) $$\zeta =\Phi -\Phi_{0},\;\text{where}\;\Phi =\frac{V_{f}(x,y,z,t)}{V(x,y,z,t)}.$$

Here $V_{f}(x,y,z,t)$ is the fluid volume and V(x,y,z,t) is the representative elementary volume centered at (x,y,z) at time t. The quantity Φ is also referred to as porosity, and $\Phi_{0}$ is the equilibrium porosity.

One can show that ζ defined above can be expressed in terms of the fluid pressure and the volumetric change of pores’ volume as

(4) $$\zeta =c_{0}p_{p}+\alpha \nabla \cdot \eta ,$$

which is the quantity appearing under the time derivative in the second equation in (2). The dependence of hydrogel’s permeability on ζ was noted in, e.g., [7,26], and it is associated with swelling of hydrogels. To describe the dependence of κ on ζ, it is common to use the Kozeny-Carman equation:

(5) $$\kappa (x,y,z,\Phi )=\kappa_{0}(x,y,z){\left(\frac{1\;-\;\Phi_{0}}{1\;-\;\Phi }\right)}^{\beta }.$$

Here the exponent β was calculated for hydrogels to be β=2/3 using geometric considerations, see [7]. The factor $\kappa_{0}$ is the reference, equilibrium permeability. In our simulations, $\kappa_{0}=\kappa_{0}(x,y,z)$ will be estimated from the micro-scale simulations using Smoothed Particle Hydrodynamics and Encoder–Decoder Convolution Neural Networks. See Section 3.3. Throughout this manuscript, we will be using either a given permeability κ(x,y,z,t), or a nonlinear permeability κ=κ(x,y,z,Φ)=κ(ζ) given by (5), rendering the Biot system (2) a *nonlinear* Biot problem.

#### Moving domain and ALE formulation.

Before we describe the coupling conditions between the fluid and poroelastic structure, we must deal with the fact that the fluid domain is moving, and the fluid equations are written in the Eulerian framework on $\Omega_{f}(t)$, while the structure equations, i.e., the Biot model, is given in the Lagrangian framework on the reference domain ${\overset{ˆ}{\Omega }}_{p}$. To deal with the motion of the fluid domain, we introduce a family of Arbitrary Lagrangian–Eulerian (ALE) mappings that map the reference fluid domain ${\overset{ˆ}{\Omega }}_{f}$ onto the current domain Ω(t), and rewrite the fluid equations in the so-called ALE form.

For this purpose, let ${\overset{ˆ}{\Omega }}_{f}\subset R^{3}$ be a fixed reference domain. We define a family of mappings:

(6) $$A_{t}^{f}:{\overset{ˆ}{\Omega }}_{f}⟶\Omega_{f}(t),\;A_{t}^{f}:x_{0}\mapsto x,$$

where x and $x_{0}$ are the coordinates in the physical domain $\Omega_{f}(t)$ and the reference domain ${\overset{ˆ}{\Omega }}_{f}$, respectively. The fluid domain velocity $w_{f}$ is given by

(7) $$w_{f}(t,\cdot )=\frac{dA_{t}^{f}}{dt}(t,A_{t}^{f}(t,\cdot {)}^{-1}).$$

Using this notation, we calculate the ALE time derivative of the fluid velocity:

(8) $${\left.\partial_{t}u_{f}\right|}_{x_{0}}=\partial_{t}u_{f}(t,x)+w_{f}(t,x)\cdot \nabla u_{f}(t,x),\;\text{for}\;x=A_{t}^{f}\left(x_{0}\right),x_{0}\in {\overset{ˆ}{\Omega }}_{f},$$

where ${\left.\partial_{t}u_{f}\right|}_{x_{0}}$ denotes the time derivative in the reference configuration ${\overset{ˆ}{\Omega }}_{f}$. The incompressible Navier–Stokes equations in ALE form are given by the following:

(9) $$\left.\begin{array}{l} \rho_{f}\left({\left.\frac{\partial u_{f}}{\partial t}\right|}_{{\overset{ˆ}{x}}_{0}}+\left(u_{f}-w_{f}\right)\cdot \nabla u_{f}\right)=\nabla \cdot \sigma_{f}\left(u_{f},p_{f}\right),\\ \nabla \cdot u_{f}=0, \end{array}\right\}\;\text{in}\;\Omega_{f}(t)\times (0,T),$$

The ALE mapping defined in terms of the fluid domain displacement $d\left(x_{0},t\right)$ is given by

(10) $$A_{t}^{f}\left(x_{0}\right)=x_{0}+d\left(x_{0},t\right),$$

where we calculate $d\left(x_{0},t\right)$ as the harmonic extension of the boundary data:

(11) $$\Delta d=0\;\text{in}\;{\overset{ˆ}{\Omega }}_{f},\;d=\eta \;\text{on}\;\overset{ˆ}{\Gamma },\;d=0\;\text{on}\;\partial {\overset{ˆ}{\Omega }}_{f}/\overset{ˆ}{\Gamma }.$$

Here ${\overset{ˆ}{\Omega }}_{f}$ denotes the reference fluid domain, and $\overset{ˆ}{\Gamma }$ is the reference fluid–structure interface.

#### The coupling conditions for the fluid poroelastic structure interaction problem.

At the fluid–structure interface Γ(t), we impose two kinematic coupling conditions, denoted by (K1) and (K2) below, and two dynamic coupling conditions, denoted by (D1) and (D2) below (see [27,28]). The coupling conditions will be stated on the reference fluid–structure interface $\overset{ˆ}{\Gamma }=\partial {\overset{ˆ}{\Omega }}_{f}\cap \partial {\overset{ˆ}{\Omega }}_{p}$. The values of the fluid velocity $u_{f}$ below are assumed at the current interface Γ(t), but evaluated on the reference configuration $\overset{ˆ}{\Gamma }$. The notation ${\left.u_{f}\right|}_{\Gamma (t)}$ on $\overset{ˆ}{\Gamma }$ used below means

(12) $${\left.u_{f}\right|}_{\Gamma (t)}=u_{f}\circ A_{t}^{f}(\overset{ˆ}{x},t),(\overset{ˆ}{x},t)\in \overset{ˆ}{\Gamma }.$$

The same holds for the normal stress $\sigma_{f}n$ on $\overset{ˆ}{\Gamma }$.

The coupling conditions are:

(K1) Continuity of normal components of fluid velocities describing fluid penetration into the poroelastic structure in the normal direction:

(13) $$u_{p}\cdot n=({\left.u_{f}\right|}_{\Gamma (t)}-\frac{\partial \eta }{\partial t})\cdot n,\;\text{on}\;\overset{ˆ}{\Gamma }\times (0,T),$$

(K2) The Beavers-Joseph-Saffman condition describing slip between the fluid and structure velocities in the tangential direction, with parameter β denoting the slip length (the inverse of which describes friction) [29,30], and J the Jacobian of the transformation between the Eulerian and Lagrangian frameworks:

(14) $$\beta \left({\left.u_{f}\right|}_{\Gamma (t)}-\frac{\partial \eta }{\partial t}\right)\cdot t_{i}=-t_{i}\cdot J\sigma_{f}n,\;\text{on}\;\overset{ˆ}{\Gamma }\times (0,T),i=1,2.$$

(D1) Continuity of dynamic pressure across the interface:

(15) $$n\cdot J\sigma_{f}n+\rho_{f}\frac{{\left.{\left|u_{f}\right|}_{\Gamma (t)}\right|}^{2}}{2}=-p_{p},\;\text{on}\;\overset{ˆ}{\Gamma }\times (0,T),$$

(D2) The balance of contact forces:

(16) $$J\sigma_{f}n-\sigma_{p}n=0,\;\text{on}\;\overset{ˆ}{\Gamma }\times (0,T).$$

#### The boundary and initial data.

The coupled FSI problem for blood plasma consists of the Navier–Stokes-Biot problem (1)–(2), with the coupling conditions (13)–(16), and the following boundary and initial data:

(17) $$u_{f}\cdot t=0,\;P_{\text{in}/\text{out}\;}(t)=p_{f}+\frac{\rho_{f}}{2}{\left|u_{f}\right|}^{2}\;\text{on}\;\Gamma_{\text{in/out}}^{f}\times (0,T)\;\eta =0\;\text{on}\;\Gamma_{\text{in}/\text{out}\;}^{f}\cup \Gamma_{\text{ext}}^{p}\times (0,T),\;\nabla p_{p}\cdot n_{p}=0\;\text{on}\;\Gamma_{\text{ext}}^{p}\times (0,T)$$

(18) $${\left.u_{f}\right|}_{t=0}=u_{f}^{0}\;\text{in}\;{\overset{ˆ}{\Omega }}_{f}(0),{\left.\;\eta \right|}_{t=0}={\left.\partial_{t}\eta \right|}_{t=0}=0\;\mathrm{in}\;{\overset{ˆ}{\Omega }}^{p},{\left.\;p_{p}\right|}_{t=0}=0\;\mathrm{in}\;{\overset{ˆ}{\Omega }}^{p}\text{.}\;$$

No-slip boundary condition for the fluid is assumed on the (remaining) rigid part of the fluid domain boundary, denoted by Γ in Figure 7. In Figure 7, we show the entire fluid and poroelastic structure domains (a 2D slice) together with their boundaries.

##### Inlet flow.

To account for the presence of four nanopore-size permeable membranes at the inlet, we use an experimentally derived relationship between flow and pressure gradient (Darcy law) through nanoporous membranes, derived in [2]. More precisely, it was demonstrated in Shuvo Roy’s lab, see [2], that silicon nanopore membranes with pore sizes of 7 nm generated a hydraulic permeability of 130 mL/h/m^2^/mmHg. More generally, the results in [2] postulate the following experimentally validated nano-macro scale Darcy relationship between the nanopore membrane parameters and the macro-scale filtration flow parameters:

(19) $$w=(12\mu hQ/nl\Delta p{)}^{\frac{1}{3}},$$

where w is the pore width, l is the pore length, h is the membrane thickness, n is the number of pores per (unit) membrane, μ is the viscosity, Q is the volumetric flow rate, and Δp is transmembrane pressure. This information was used to find the **macro-scale inlet flow based on the micro-scale parameters that are specific to membrane structure**. Namely, given the inlet pressure $P_{\mathrm{in}}(t)$, the filtration flow through the membranes at time $t^{n+1}$ was calculated via

(20) $$u^{n+1}\cdot n=\frac{w^{2}}{12\mu h}\left(P_{\mathrm{in}}-p_{\mathrm{gasket}}^{n}\right)\;\text{on}\;\Gamma_{\mathrm{in}}^{f},$$

where $p_{\mathrm{gasket}}^{n}$ is the gasket pressure at time $t^{n}$. Here we used that the total pore area for each membrane is given by nlw.

In addition to the micro-macro scale relationship (19) the work published in [2] demonstrated the feasibility of silicon nanopore membranes for immunoisolation, by measuring the selectivity against transport of cytokines and small molecules using the pressure-driven ultrafiltration system.

Table 1 shows the parameter values used in this FSI model.

#### Energy estimate.

To show that the coupled problem is well-defined in terms of having a bounded energy, which is related to stability, we show below that the total energy of the problem, and the total dissipation, are bounded by a constant that only depends on the initial and boundary data. In the energy estimate below we assume that κ=κI, where κ may be a nonlinear bounded function of the fluid content $\zeta =\Phi -\Phi_{0}$ as in (5), or a bounded function of (x,y,z,t). More precisely, using the approaches similar to the proof of Theorem 3.1 in [31] one can show that the following energy estimate holds.

#### Theorem 1.

*The energy of the coupled Navier–Stokes-Biot problem* (1)–(2), (13)–(18), *satisfies the following inequality:*

(21) $$\frac{d}{dt}E(t)+D(t)\leq C(t),$$

where E(t) denotes the kinetic energy of fluid and the kinetic and elastic energy of the poroelastic structure:

(22) $$E(t)=\frac{\rho_{f}}{2}{\left\|u_{f}\right\|}_{L^{2}\left(\Omega_{f}(t)\right)}^{2}+\frac{\rho_{p}}{2}{\left\|\partial_{t}\eta \right\|}_{L^{2}\left({\overset{ˆ}{\Omega }}_{p}\right)}^{2}+\frac{c_{0}}{2}{\left\|p_{p}\right\|}_{L^{2}\left({\overset{ˆ}{\Omega }}_{p}\right)}^{2}+\mu_{p}\|D(\eta ){\|}_{L^{2}\left({\overset{ˆ}{\Omega }}_{p}\right)}^{2}+\frac{\lambda_{p}}{2}\|\nabla \cdot \eta {\|}_{L^{2}\left({\overset{ˆ}{\Omega }}_{p}\right)}^{2}$$

and D(t) denotes the total dissipation:

(23) $$D(t)=\mu_{f}{\left\|D\left(u_{f}\right)\right\|}_{L^{2}\left(\Omega_{f}(t)\right)}^{2}+{\left\|\kappa^{\frac{1}{2}}\nabla p_{p}\right\|}_{L^{2}\left({\overset{ˆ}{\Omega }}_{p}\right)}^{2}+\beta {\left\|\left(u_{f}-\partial_{t}\eta \right)\cdot t\right\|}_{L^{2}(\overset{ˆ}{\Gamma }{)}^{'}}^{2}$$

and C(t) depends only on the initial and boundary data.

### 2.2. Coupled Models for Oxygen Concentration

We present two models for oxygen concentration, both defined on moving domains obtained from the fluid–structure interaction problem discussed above. One is an advection-diffusion equation for the concentration $C_{f}(x,t)$ of oxygen in the fluid channel/gasket, defined on $\Omega_{f}(t)$, and the other is a nonlinear advection–reaction–diffusion equation for oxygen concentration in the scaffold, $C_{p}(x,t)$, defined on $\Omega_{p}(t)$. The two models are coupled at the interface Γ(t) separating the gasket flow from the poroelastic scaffold. The oxygen concentration models are coupled to the fluid–structure interaction problem above via the advection velocity obtained from the FSI problem above, and via the fluid domain motion. This is a one-way coupling because nothing from the problem for oxygen concentration influences the solution of the FSI problem.

The advection-diffusion for the concentration $C_{f}$ of **oxygen in the fluid channel/gasket** written in conservation form reads:

(24) $$\frac{\partial C_{f}}{\partial t}+\nabla \cdot \left(u_{f}C_{f}\right)-\nabla \cdot \left(D_{f}\nabla C_{f}\right)=0,\;\text{in}\;\Omega_{f}(t)\times (0,T),$$

where the advection velocity $u_{f}$ is given by the solution of the Navier–Stokes equations in domain $\Omega_{f}(t)$. Here, $D_{f}$ is the free oxygen diffusion coefficient $\left(D_{f}=2.41\times 10^{-5}{cm}^{2}s^{-1}\right)$ in blood [32]. This model is coupled to the gasket fluid flow model via the fluid advection velocity $u_{f}$ obtained from the Navier–Stokes Equation (1), and via the fluid domain motion $\Omega_{f}(t)$.

The nonlinear advection–reaction–diffusion equation for concentration $C_{p}$ of **oxygen in the hydrogel** is defined on the *moving domain* $\Omega_{p}(t)$. Thus, the advection velocity in this case must be given by the *sum* of the *relative* filtration velocity $u_{p}$, obtained from the Biot Equation (2), *plus* the velocity of the motion of the hydrogel skeleton:

(25) $$w_{p}=\partial_{t}\eta .$$

Therefore, we introduce

(26) $${\tilde{u}}_{p}=u_{p}+w_{p}$$

and write the equation for oxygen concentration $C_{p}$ in the poroelastic hydrogel on the moving domain $\Omega_{p}(t)$ in conservation form as follows:

(27) $$\frac{\partial C_{p}}{\partial t}+\nabla \cdot \left({\tilde{u}}_{p}C_{p}\right)=\nabla \cdot \left(D_{p}\nabla C_{p}\right)+R_{max}\frac{C_{p}}{C_{p}\;+\;C_{MM}}ℋ\left(C_{p}>C_{cr}\right),\;\text{in}\;{\overset{ˆ}{\Omega }}_{p}(t)\times (0,T),$$

where $R_{\max}$ is the maximum oxygen consumption rate, $C_{MM}$ is the Michaelis–Menten constant corresponding to the oxygen concentration where consumption drops to 50% of its maximum [8], $C_{cr}$ is the critical oxygen concentration below which necrosis is assumed to occur after a sufficiently long exposure, and ℋ is the Heaviside step-down function to account for the ceasing of consumption in those parts of the tissue where the oxygen concentration fell below a critical concentration $C_{cr}$ [8,33]. $D_{p}$ is the diffusion coefficient of oxygen, whose value has been estimated for rat pancreatic islets to be 1.3 × 10^−9^ cm^2^ s^−1^.

#### The ALE formulation of the oxygen concentration models.

Before we rewrite Equations (24) and (27) in ALE form, we recall that the advection in (27) is driven by the plasma filtration velocity ${\tilde{u}}_{p}=u_{p}+w_{p}$, where $u_{p}$ is the relative filtration velocity obtained from the Biot model defined on ${\overset{ˆ}{\Omega }}_{p}$. Namely, $u_{p}$ denotes the composite function between the relative filtration velocity defined on ${\overset{ˆ}{\Omega }}_{p}$ and the inverse of the ALE mapping ${𝒜}_{t}^{p}:{\overset{ˆ}{\Omega }}_{p}\to \Omega_{p}(t)$ associated with the motion of the poroelastic matrix ${𝒜}_{t}^{p}:x_{0}\mapsto x_{0}+\eta \left(x_{0},t\right)$. Therefore, ${\tilde{u}}_{p}=u_{p}+w_{p}$, where $u_{p}={\left.{𝒜}_{t}^{p}\circ u_{p}\right|}_{\overset{ˆ}{\Omega }}\left({\left({𝒜}_{t}^{p}\right)}^{-1}x,t\right)$ and $w_{p}=\partial_{t}\eta$.

Equation (24), defined on the current moving domain $\Omega_{f}(t)\times (0,\;T)$ in ALE form reads:

(28) $${\left.\frac{\partial C_{f}}{\partial t}\right|}_{x_{0}}-\nabla \cdot \left(D_{f}\nabla C_{f}\right)+\left(u_{f}-w_{f}\right)\cdot \nabla C_{f}=0,$$

where $w_{f}$ is the fluid domain velocity.

Equation (27), defined on the current moving domain $\Omega_{p}(t)\times (0,T)$, in ALE form reads:

(29) $${\left.\frac{\partial C_{p}}{\partial t}\right|}_{x_{0}}-w_{p}\cdot \nabla C_{p}+\nabla \cdot \left({\tilde{u}}_{p}C_{p}\right)-\nabla \cdot \left(D_{p}\nabla C_{p}\right)=R_{max}\frac{C_{p}}{C_{p}\;+\;C_{MM}}\delta \left(C_{p}>C_{cr}\right).$$

Notice that the time derivatives of oxygen concentration ${\left.\frac{\partial C_{f}}{\partial t}\right|}_{x_{0}}$ and ${\left.\frac{\partial C_{p}}{\partial t}\right|}_{x_{0}}$ are evaluated on the corresponding reference domains and then mapped back onto the corresponding moving domains where the ALE equations are defined.

In conservation form, Equations (28) and (29) above read as follows.

**On** $\Omega_{f}(t)\times (0,T)$ **we have:**

(30) $${\left.\frac{\partial C_{f}}{\partial t}\right|}_{x_{0}}+\nabla \cdot \left[\left(u_{f}-w_{f}\right)C_{f}-D_{f}\nabla C_{f}\right]+\left(\nabla \cdot w_{f}\right)C_{f}=0,$$

where ALE velocity $w_{f}$ is given by (7).

**On** $\Omega_{p}(t)\times (0,T)$ **we have:**

(31) $${\left.\frac{\partial C_{p}}{\partial t}\right|}_{x_{0}}+\nabla \cdot \left[\left({\tilde{u}}_{p}-w_{p}\right)C_{p}-D_{p}\nabla C_{p}\right]+\left(\nabla \cdot w_{p}\right)C_{p}=R_{max}\frac{C_{p}}{C_{p}\;+\;C_{MM}}ℋ\left(C_{p}>C_{cr}\right),$$

where $w_{p}$ is given by (25).

#### Coupling conditions.

Two Equations (28) and (29) are coupled across the moving interface Γ(t) via the following two coupling conditions, describing continuity of oxygen concentration across the interface and continuity of total oxygen fluxes:

$$\left.\begin{array}{c} C_{f}=C_{p}\\ \left[\left(u_{f}-w_{f}\right)C_{f}-D_{f}\nabla C_{f}\right]\cdot n_{f}=\left[\left({\tilde{u}}_{p}-w_{p}\right)C_{p}-D_{p}\nabla C_{p}\right]\cdot n_{f} \end{array}\right\}\;\text{on}\;\Gamma (t)\times (0,T).$$

Because of the kinematic coupling condition at the interface $u_{p}\cdot n=\left({\left.u_{f}\right|}_{\Gamma (t)}-\frac{\partial \eta }{\partial t}\right)\cdot n$ and the fact that $w_{f}=\partial_{t}\eta$ at the interface and that ${\tilde{u}}_{p}-w_{p}=u_{p}$ is the relative filtration velocity with respect to the motion of the poroelastic matrix, after using the continuity of concentrations $C_{f}=C_{p}$ at the interface, one obtains the following coupling conditions:

(32) $$\left.\begin{array}{c} C_{f}=C_{p}\\ D_{f}\nabla C_{f}\cdot n_{f}=D_{p}\nabla C_{p}\cdot n_{f} \end{array}\right\}\;\text{on}\;\Gamma (t)\times (0,T).$$

#### Initial and boundary conditions.

The coupled system (28), (29) and (32) is supplemented with the following initial and boundary conditions:

(33) $$C_{f}=C_{f,0},\;\text{in}\;\Omega_{f}\times \{t=0\},\;C_{p}=C_{p,0},\;\text{in}\;\Omega_{p}\times \{t=0\}$$

(34) $$C_{f}=C_{\mathrm{inlet}},\;\mathrm{on}\;\Gamma_{\mathrm{in}\;}^{f},\;D_{f}\nabla C_{f}\cdot n_{f}=0,\;\mathrm{on}\;\Gamma_{\mathrm{out}}^{f},\;D_{p}\nabla C_{p}\cdot n_{p}=0,\;\mathrm{on}\;\Gamma_{\mathrm{ext}}^{p},$$

where $C_{f,0}$ and $C_{p,0}$ are initial concentrations in the fluid channel and hydrogel, respectively, which are assumed to be zero. Here, $\Gamma_{\mathrm{in}}^{f}$ and $\Gamma_{\mathrm{out}}^{f}$ denote the fluid channel inlet and outlet, and $\Gamma_{\mathrm{ext}}^{f}$ denotes external sidewalls of the hydrogel, where we assume that oxygen cannot penetrate the external sidewalls. All the parameters used in the simulations are obtained from [8,33] and reported in Table 2.

#### Energy Estimate.

The following energy estimate shows that the coupled advection–reaction–diffusion problem for oxygen concentration (30)–(34) is well-defined in terms of having a bounded energy.

#### Theorem 2.

*Let* ${\tilde{u}}_{p}\in L^{2}\left(0,T;L^{2}\left(\Omega_{p}(t)\right)\right)$ *and* $\nabla \cdot {\tilde{u}}_{p}\in L^{2}\left(0,T;L^{2}\left(\Omega_{p}(t)\right)\right)$. *Then, there exist* $K(t)>0,\;\tilde{C}(t)>0$, *and* ${\tilde{D}}_{p}>0$ *such that the coupled advection–reaction–diffusion problem* (30)–(34) *satisfies the following energy estimate:*

(35) $${\left\|C_{f}\right\|}_{L^{2}\left(\Omega_{f}(t)\right)}^{2}+{\left\|C_{p}\right\|}_{L^{2}\left(\Omega_{p}(t)\right)}^{2}+2\int_{0}^{t}\;{\left\|D_{f}\nabla C_{f}\right\|}_{L^{2}\left(\Omega_{f}(\tau )\right)}^{2}d\tau +2\int_{0}^{t}\;{\left\|{\tilde{D}}_{p}\nabla C_{p}\right\|}_{L^{2}\left(\Omega_{p}(\tau )\right)}^{2}d\tau$$

(36) $$\leq K(t)e^{2\int_{0}^{t}\;\tilde{C}(t)},$$

*where* $\tilde{C}(t)$ *depends on* ${\left\|\nabla \cdot {\tilde{u}}_{p}\right\|}_{L^{2}\left(\Omega_{p}(t)\right)}^{2}$, *and* K(t) *depends on the initial data and on* $\tilde{C}(t)$.

The proof of this energy estimate is similar to the proof of Theorem 3.2 in [31].

## 3. Discretized Problems and Numerical Schemes

We use Finite Element Method-based schemes to solve the FSI problem (1)–(17) and the coupled advection–reaction–diffusion problem for oxygen concentration (28), (29) and (32) on moving domains. For the FSI problem we introduce a new scheme based on Nitsche’s method, which improves the already existing schemes in the following two ways: the proposed method has accuracy *higher than 1st order* and it solves a *nonlinearly coupled* problem between the Navier–Stokes equations defined on *moving domain* and the Biot equations. We also prove that this new numerical scheme is *unconditionally stable*.

To couple the macro-scale simulations with the micro-scale information, we use Smoothed Particle Hydrodynamics (SPH) simulations. In particular, we recover the information about hydraulic permeability locally, for every small “control” volume, for a given hydrogel structure, and use it to recover the global, macro-scale permeability tensor κ. To avoid time-consuming and computationally expensive SPH simulations for every new hydrogel structure, we use Convolution Neural Networks, trained on a set of our synthetic data (calculated off line), to obtain κ for new hydrogel structures which are either generated synthetically (using our numerical simulations with random pore distributions), or for the actual hydrogel structures that can be imaged using high precision laser scanning confocal microscopy.

Details are presented next.

### 3.1. Discretization of the Fluid-Structure Interaction Problem

We discretize and solve the FSI problem involving poroelastic structure in a monolithic way, using the refactorized Cauchy’s ‘θ-like’ method for the time discretization (see [35]), which is equivalent to the midpoint method when $\theta =\frac{1}{2}$. In this case the method is conservative and second-order accurate in time. To discretize the problem in space, we use $P_{2}-P_{1}$ elements for the fluid velocity and pressure, and $P_{2}$ elements for the structure displacement and velocity, and $P_{2}-P_{1}$ for the Biot filtration velocity and pressure. Note, again, that the fluid–structure interaction problem and the advection–reaction–diffusion problem are defined on moving domains.

To enforce the coupling conditions, we use Nitsche’s method, similar to [27]. In [27], the Nitsche’s method was first-order accurate in time, and it was applied to solve a Stokes–Biot-coupled problem where the coupling was assumed across a *fixed* interface, i.e., linear coupling. In [31], this method was extended to a Navier–Stokes/Biot-coupled problem with the coupling assumed across the *current, moving interface*, i.e., nonlinear coupling, with first-order accuracy in time. In the present work, we extend this method to improve the time-accuracy to second-order, and we prove that the proposed method is *unconditionally stable*. Before we present the discretized problem, we first state the continuous weak formulation.

#### Weak formulation.

We solve the coupled FSI problem (1)–(2), with the coupling conditions (13)–(16), and the initial and boundary conditions (18) and (17) in mixed formulation (see, e.g., [15,27,36] for the Stokes-Biot mixed formulation). The corresponding continuous **weak formulation** is given by the following:

(37) $$\begin{array}{l} \;-\rho_{f}\int_{0}^{T}\;\int_{\Omega_{f}(t)}\;u_{f}\cdot \partial_{t}\phi_{f}dxdt+\rho_{f}\int_{0}^{T}\;\int_{\Omega_{f}(t)}\;\left(u_{f}\cdot \nabla \right)u_{f}\cdot \phi_{f}dxdt-\rho_{p}\int_{0}^{T}\;\int_{{\overset{ˆ}{\Omega }}_{p}}\;\dot{\eta }\cdot \partial_{t}\xi_{p}dxdt\\ \;+2u_{f}\int_{0}^{T}\;\int_{\Omega_{f}(t)}\;D\left(u_{f}\right):D\left(\phi_{f}\right)dxdt+\int_{0}^{T}\;\int_{{\overset{ˆ}{\Omega }}_{p}}\;2\mu_{s}D(\eta ):D\left(\xi_{p}\right)+\lambda_{p}(\nabla \cdot \eta )\left(\nabla \cdot \xi_{p}\right)dxdt\\ \;-\alpha \int_{0}^{T}\;\int_{{\overset{ˆ}{\Omega }}_{p}}\;p_{p}\left(\nabla \cdot \xi_{p}\right)dxdt-\rho_{p}\int_{0}^{T}\;\int_{{\overset{ˆ}{\Omega }}_{p}}\;\eta \cdot \partial_{t}\varphi_{p}dxdt-\rho_{p}\int_{0}^{T}\;\int_{{\overset{ˆ}{\Omega }}_{p}}\;\dot{\eta }\cdot \varphi_{p}dxdt\\ \;+c_{0}\int_{0}^{T}\;\int_{{\overset{ˆ}{\Omega }}_{p}}\;p_{p}\partial_{t}\psi_{p}dxdt+\int_{0}^{T}\;\int_{{\overset{ˆ}{\Omega }}_{p}}\;\left(\nabla \cdot u_{p}\right)\psi_{p}dxdt+\alpha \int_{0}^{T}\;\int_{{\overset{ˆ}{\Omega }}_{p}}\;\left(\nabla \cdot \partial_{t}\eta \right)\psi_{p}dxdt\\ \;+\int_{0}^{T}\;\int_{{\overset{ˆ}{\Omega }}_{p}}\;\frac{1}{\kappa }u_{p}\cdot \phi_{p}dxdt-\int_{0}^{T}\;\int_{{\overset{ˆ}{\Omega }}_{p}}\;p_{p}\left(\nabla \cdot \phi_{p}\right)dxdt+\int_{0}^{T}\;I_{\overset{ˆ}{\Gamma }}^{bdry}(t)dt\\ \;=\int_{0}^{T}\;\int_{\Gamma_{in}^{f}}\;P_{in}(t)\left(\phi_{f}\cdot n\right)dSdt-\int_{0}^{T}\;\int_{\Gamma_{out}^{f}}\;P_{out}(t)\left(\phi_{f}\cdot n\right)dSdt+\int_{0}^{T}\;\int_{{\overset{ˆ}{\Omega }}_{p}}\;\left(F^{s}\cdot \xi_{p}+F_{p}\psi^{p}\right)dxdt\\ \;+{\left.\int_{\Omega_{f}(t)}\;\left(u_{f}\cdot \phi_{f}\right)\right|}_{t=0}dx+{\left.\int_{{\overset{ˆ}{\Omega }}_{p}}\;\left(\dot{\eta }\cdot \xi_{p}\right)\right|}_{t=0}dx+{\left.\int_{{\overset{ˆ}{\Omega }}_{p}}\;\left(\eta \cdot \varphi_{p}\right)\right|}_{t=0}dx+{\left.\int_{{\overset{ˆ}{\Omega }}_{p}}\;\left(p_{p}\partial_{t}\psi_{p}\right)\right|}_{t=0}dx \end{array}$$

where

(38) $$I_{\overset{ˆ}{\Gamma }}^{bdry}(t)=\beta \sum_{i=1,2}\int_{\overset{ˆ}{\Gamma }}\left(t_{i}\cdot \left(u_{f}-\partial_{t}\eta \right)\right)\left(t_{i}\cdot \phi_{f}-t_{i}\cdot \xi_{p}\right)dS+\int_{\overset{ˆ}{\Gamma }}\frac{\rho_{f}}{2}{\left|u_{f}\right|}^{2}\left(\phi_{p}\cdot n\right)dS.$$

The vectors $t_{i}$, i=1,2 are two linearly independent tangent vectors at the interface. The kinematic coupling condition (13) must be included in the solution and test function spaces, and is numerically enforced by Nitsche’s penalization [27].

The function spaces for the fluid velocity are denoted by $V^{f}(t)$ and $V_{\text{div}}^{f}(t)$ below, and the function space for the pressure is denoted by $Q^{f}(t)$, all defined on $\Omega_{f}(t)$. The functions spaces for the filtration velocity and fluid pore pressure are denoted by $V^{p}$ and $Q^{p}$ below, both defined on ${\overset{ˆ}{\Omega }}_{p}$, and the function spaces for the structure displacement and velocity are denoted by $X^{p}$ and ${\dot{X}}^{p}$, both defined on ${\overset{ˆ}{\Omega }}_{p}$. The spaces are defined as follows:

(39) $$\begin{array}{l} V^{f}(t)=\left\{\mathrm{vs}.\in {\left[H^{1}\left(\Omega_{f}(t)\right)\right]}^{d}|\mathrm{vs}.\cdot t=0\;\mathrm{on}\;\Gamma_{\mathrm{in}}^{f}\cup \Gamma_{\mathrm{out}}^{f},\;\mathrm{vs}.=0\;\mathrm{on}\;\Gamma_{\mathrm{ext}}^{f}\right\},\\ V_{\text{div}\;}^{f}(t)=\left\{\mathrm{vs}.\in V^{f}(t)|\nabla \cdot \mathrm{vs}.=0\right\},\;Q^{f}(t)=L^{2}\left(\Omega_{f}(t)\right),\\ V^{p}=\left\{u_{p}\in H\left(div,{\overset{ˆ}{\Omega }}_{p}\right)|u_{p}\cdot n_{p}=0\right\}=\left\{u_{p}\in {\left[L^{2}\left({\overset{ˆ}{\Omega }}_{p}\right)\right]}^{d}|\nabla \cdot u_{p}\in L^{2}\left({\overset{ˆ}{\Omega }}_{p}\right),u_{p}\cdot n_{p}=0\right\},\\ Q^{p}=L^{2}\left({\overset{ˆ}{\Omega }}_{p}\right),\;X^{p}={\left[H^{1}\left({\overset{ˆ}{\Omega }}_{p}\right)\right]}^{d},\;{\dot{X}}^{p}={\left[L^{2}\left({\overset{ˆ}{\Omega }}_{p}\right)\right]}^{d}. \end{array}$$

We further introduce the following Bochner spaces:

$$\begin{array}{l} W^{f}=L^{\infty }\left(0,T;L^{2}\left(\Omega_{f}(t)\right)\right)\cap L^{2}(0,T;V_{\text{div}\;}^{f}(t)),\;{𝒳}^{p}=L^{\infty }\left(0,T;X^{p}\right),\\ {\dot{𝒳}}^{p}=L^{\infty }(0,T;{\dot{X}}^{p}),{𝒬}^{p}=L^{\infty }(0,T;L^{2}({\overset{ˆ}{\Omega }}_{p}))\cap L^{2}\left(0,T;Q^{p}\right),\;{𝒱}^{p}=L^{2}\left(0,T;V^{p}\right). \end{array}$$

The function space for the solution of the FSI problem is then defined by:

$$𝒮=\left\{\left(u_{f},u_{p},p_{p},\eta ,\dot{\eta }\right)\in (W^{f}\times {𝒱}^{p}\times {𝒬}^{p}\times {𝒳}^{p}\times {\dot{𝒳}}^{p})|u_{p}\cdot n=({\left.u_{f}\right|}_{\Gamma (t)}-\partial_{t}\eta )\cdot n\;\mathrm{on}\;\overset{ˆ}{\Gamma }\right\},$$

with the corresponding test space:

$$\begin{array}{c} 𝒯=\left\{\left(\phi_{f},\phi_{p},\psi_{p},\varphi_{p},\xi_{p}\right)\right.\;\in C_{c}^{1}([0,T);V_{div}^{f}(t)\times V^{p}\times Q^{p}\times X^{p}\times {\dot{X}}^{p})\\ \left.|\phi_{p}\cdot n=({\left.\phi_{f}\right|}_{\Gamma (t)}-\partial_{t}\varphi_{p})\cdot n\;\mathrm{on}\;\overset{ˆ}{\Gamma }\right\}. \end{array}$$

In this case, instead of the homogeneous Dirichlet boundary condition for Darcy pressure on $\Gamma_{\mathrm{ext}}$, we impose the condition on Darcy velocity $u_{p}\cdot n=0$ on $\Gamma_{\mathrm{ext}}$. We present the discretized problem based on this weak formulation next.

#### Discretization.

Let the shape of $\Omega_{f}(t)$ and ${\overset{ˆ}{\Omega }}_{p}$ be polygons/tetrahedra. Denote by h the characteristic mesh size and suppose that $T_{h}^{f}$ and $T_{h}^{p}$ are the uniform conforming triangulation of $\Omega_{f}(t)$ and ${\overset{ˆ}{\Omega }}_{p}$, respectively. Based on the meshes, we define the finite element spaces $V_{h}^{f}\subset V^{f}$, $Q_{h}^{f}\subset Q^{f}$, $V_{h}^{p}\subset V^{p}$, $Q_{h}^{p}\subset Q^{p}$, $X_{h}^{p}\subset X^{p}$, ${\dot{X}}_{h}^{p}\subset {\dot{X}}^{p}$ for the spatial discretization, where the function spaces $V^{f}$, $V^{p}$, $Q^{p}$, $X^{p}$, and ${\dot{X}}^{p}$ are defined in (39).

Below, we will use Nitsche’s method to enforce the coupling conditions at the discrete level. In particular, in Step 1 below, we introduce a penalty parameter $\gamma_{f}>0$ to enforce the kinematic coupling condition. In Step 1 below we also introduce a flag ς∈{1,0,-1} which determines whether a symmetric, incomplete, or skew-symmetric formulation is adopted [27] in the formulation.

More precisely, let $t^{n}=n\Delta t$ for n=0,…,N, where Δt denotes the time step, and $t^{n+\theta }=t^{n}+\theta \Delta t$, for any $\theta \in \left[\frac{1}{2},1\right]$, and for all n≥0. The fully discretized coupled FSI problem in ALE form and the numerical scheme to solve it, are given by the following three steps.

Step 1: Given $u_{f,h}^{n}$, $w^{n}$, ${\dot{\eta }}_{h}^{n}$, $\eta_{h}^{n}$, $p_{p,h}^{n}$, compute $u_{f,h}^{n+\theta }$, $p_{f,h}^{n+\theta }$, ${\dot{\eta }}_{h}^{n+\theta }$, $u_{p,h}^{n+\theta }$, $p_{p,h}^{n+\theta }$, such that

(40) $$\begin{array}{l} \rho_{f}\int_{\Omega_{f}^{n}}\;\frac{u_{f,h}^{n+\theta }-u_{f,h}^{n}}{\theta \Delta t}\cdot \phi_{f,h}dx+\rho_{f}\int_{\Omega_{f}^{n}}\;\left(\left(u_{f,h}^{n}-w^{n}\right)\cdot \nabla \right)u_{f,h}^{n+\theta }\cdot \phi_{f,h}dx\\ \;+\int_{\Omega_{f}^{n}}\;\psi_{f,h}\nabla \cdot u_{f,h}^{n+\theta }dx-\int_{\Omega_{f}^{n}}\;p_{f,h}^{n+\theta }\nabla \cdot \phi_{f,h}dx+2\mu_{f}\int_{\Omega_{f}^{n}}\;D\left(u_{f,h}^{n+\theta }\right)\;:D\left(\phi_{f,h}\right)dx\\ \;+\rho_{p}\int_{{\overset{ˆ}{\Omega }}_{p}}\;\frac{{\dot{\eta }}_{h}^{n+\theta }-{\dot{\eta }}_{h}^{n}}{\theta \Delta t}\cdot \xi_{p,h}dx+2\mu_{s}\int_{{\overset{ˆ}{\Omega }}_{p}}\;D\left(\theta \Delta t{\dot{\eta }}_{h}^{n+\theta }+\eta_{h}^{n}\right)\;:D\left(\xi_{p,h}\right)dx\\ \;+\lambda_{p}\int_{{\overset{ˆ}{\Omega }}_{p}}\;\left(\nabla \cdot \left(\theta \Delta t{\dot{\eta }}_{h}^{n+\theta }+\eta_{h}^{n}\right)\right)\left(\nabla \cdot \xi_{p,h}\right)dx-\alpha \int_{{\overset{ˆ}{\Omega }}_{p}}\;p_{p,h}^{n+\theta }\left(\nabla \cdot \xi_{p,h}\right)dx\\ \;+c_{0}\int_{{\overset{ˆ}{\Omega }}_{p}}\;\frac{p_{p,h}^{n+\theta }-p_{p,h}^{n}}{\theta \Delta t}\psi_{p,h}dx+\alpha \int_{{\overset{ˆ}{\Omega }}_{p}}\;\left(\nabla \cdot {\dot{\eta }}_{h}^{n+\theta }\right)\psi_{p,h}dx+\int_{{\overset{ˆ}{\Omega }}_{p}}\;\left(\nabla \cdot u_{p,h}^{n+\theta }\right)\psi_{p,h}dx\\ \;+\int_{{\overset{ˆ}{\Omega }}_{p}}\;{\left(\kappa^{n}\right)}^{-1}u_{p,h}^{n+\theta }\cdot \phi_{p,h}dx-\int_{{\overset{ˆ}{\Omega }}_{p}}\;p_{p,h}^{n+\theta }\left(\nabla \cdot \phi_{p,h}\right)dx-I_{h}^{*,n+\theta }\\ \;=\int_{\Gamma_{in}^{f}}\;P_{in}^{n+\theta }\left(\phi_{f,h}\cdot n\right)dS-\int_{\Gamma_{\mathrm{out}}^{f}}\;P_{\mathrm{out}}^{n+\theta }\left(\phi_{f,h}\cdot n\right)dS, \end{array}$$

where

$$I_{h}^{*,n+\theta }={\left[I_{\overset{ˆ}{\Gamma }}^{bdry}\right]}^{n+\theta }+{\left[I_{\overset{ˆ}{\Gamma }}^{Nitsche}\right]}^{n+\theta },$$

with

(41) $$\begin{array}{l} {\left[I_{\overset{ˆ}{\Gamma }}^{bdry}\right]}^{n+\theta }\;:=\int_{\overset{ˆ}{\Gamma }}\;\left(n\cdot \sigma_{f}n_{h}^{n+\theta }\right)\left(\phi_{f,h}\cdot n-\xi_{p,h}\cdot n-\phi_{p,h}\cdot n\right)dS\\ \;-\beta \int_{\overset{ˆ}{\Gamma }}\;\left(u_{f,h}^{n+\theta }-{\dot{\eta }}_{h}^{n+\theta }\right)\cdot t\left(\phi_{f,h}-\xi_{p,h}\right)\cdot tdS\\ \;+\frac{\rho_{f}}{2}\int_{\overset{ˆ}{\Gamma }}\;{\left|u_{f}^{n}\right|}^{2}\left(\phi_{p,h}\cdot n^{n+\theta }\right)dS \end{array}$$

$$\begin{array}{l} {\left[I_{\overset{ˆ}{\Gamma }}^{Nitsche}\right]}^{n+\theta }\;:=-\int_{\overset{ˆ}{\Gamma }}\;\gamma_{f}\mu_{f}h^{-1}\left[\left(u_{f,h}^{n+\theta }-u_{p,h}^{n+\theta }-{\dot{\eta }}_{h}^{n+\theta }\right)\cdot n\left(\phi_{f,h}-\xi_{p,h}-\phi_{p,h}\right)\cdot n\right]dS\\ \;+\int_{\overset{ˆ}{\Gamma }}\;n\cdot J\sigma_{f}\left(s\phi_{f,}-\psi_{f}\right)n\left(u_{f,h}^{n+\theta }-u_{p,h}^{n+\theta }-{\dot{\eta }}_{h}^{n+\theta }\right)\cdot ndS. \end{array}$$

From ${\dot{\eta }}_{h}^{n+\theta }$ we calculate $\eta_{h}^{n+\theta }$ using:

$${\dot{\eta }}_{h}^{n+\theta }=\frac{\eta_{h}^{n+\theta }-\eta_{h}^{n}}{\theta \Delta t}.$$

Step 2: Compute $u_{f,h}^{n+1}$, ${\dot{\eta }}_{h}^{n+1}$, $\eta_{h}^{n+1}$, $p_{p,h}^{n+1}$, as

$$\begin{array}{llll} u_{f,h}^{n+1}\;=\frac{1}{\theta }u_{f,h}^{n+\theta }-\frac{1-\theta }{\theta }u_{f,h}^{n} & \; & & \;\mathrm{in}\;\Omega_{f}^{n},\\ \eta_{h}^{n+1}\;=\frac{1}{\theta }\eta_{h}^{n+\theta }-\frac{1-\theta }{\theta }\eta_{h}^{n} & \; & & \;\mathrm{in}\;{\overset{ˆ}{\Omega }}_{p},\\ {\dot{\eta }}_{h}^{n+1}\;=\frac{1}{\theta }{\dot{\eta }}_{h}^{n+\theta }-\frac{1-\theta }{\theta }{\dot{\eta }}_{h}^{n} & \; & & \;\mathrm{in}\;{\overset{ˆ}{\Omega }}_{p},\\ p_{p,h}^{n+1}\;=\frac{1}{\theta }p_{p,h}^{n+\theta }-\frac{1-\theta }{\theta }p_{p,h}^{n} & \; & & \;\mathrm{in}\;{\overset{ˆ}{\Omega }}_{p}. \end{array}$$

Step 3: Update the fluid mesh by solving the following problem

(42) $$\begin{array}{llll} -\Delta \eta_{f}^{n+1}\;=0 & \;\;\;\;\;\;\;\; & & \mathrm{in}\;{\overset{ˆ}{\Omega }}_{f}\\ \eta_{f}^{n+1}\;=0 & \;\;\;\;\;\;\;\; & & \mathrm{on}\;{\overset{ˆ}{\Gamma }}_{\text{in/out}}^{f},\\ \eta_{f}^{n+1}\;=\eta^{n+1} & \;\;\;\;\;\;\;\; & & \mathrm{on}\;\overset{ˆ}{\Gamma },\\ w^{n+1}\;=\frac{\eta_{f}^{n+1}-\eta_{f}^{n}}{\theta \Delta t} & \;\;\;\;\;\;\;\; & & \mathrm{in}\;{\overset{ˆ}{\Omega }}_{f},\\ \Omega_{f}^{n+1}\;=\left(I+\eta_{f}^{n+1}\right){\overset{ˆ}{\Omega }}_{f}. \end{array}$$

We note that in Step 1, the permeability tensor $\kappa^{n}$ can be a function of the fluid content $\zeta =c_{0}p_{p}+\alpha \nabla \cdot \eta$ in which case

(43) $$\kappa^{n}=\kappa \left(c_{0}p_{p}^{n}+\alpha \nabla \cdot \eta^{n}\right)I.$$

The approximations of the solution $\left(u_{f,h}^{n+1},p_{f,h}^{n+1},u_{p,h}^{n+1},p_{p,h}^{n+1},\eta_{h}^{n+1},{\dot{\eta }}_{h}^{n+1}\right)$ belong to $V_{h}^{f}\times Q_{h}^{f}\times V_{h}^{p}\times Q_{h}^{p}\times X_{h}^{p}\times {\dot{X}}_{h}^{p}$, with the corresponding test functions $\left(\phi_{f,h},\psi_{f,h},\phi_{p,h},\psi_{p,h},\varphi_{p,h},\xi_{p,h}\right)\;\in V_{h}^{f}\times Q_{h}^{f}\times V_{h}^{p}\times Q_{h}^{p}\times X_{h}^{p}\times {\dot{X}}_{h}^{p}$. We solve the monolithic problem with a preconditioner obtained using a loosely coupled scheme, as reported in [27].

#### Stability of the FSI scheme

The focus of this section is on the stability analysis of method (40)–(42). As it is often the case, the stability analysis is presented for the linearized domain motion case (fixed fluid domain), assuming laminar flow and small interface deformation. In this case, the coupling condition (15) reduces to

(44) $$n\cdot \sigma_{f}n=-p_{p}\;\mathrm{on}\;\overset{ˆ}{\Gamma }\times (0,T).$$

We show that our proposed numerical scheme (40)–(42) written in terms of Nitsche’s method applied to the Navier–Stokes/Biot coupled problem, based on the refactorized Cauchy’s ‘θ-like’ method, is **unconditionally stable** provided $\theta \in \left[\frac{1}{2},1\right]$ and the Nitsche penalty parameter $\gamma_{f}$ satisfies a certain condition, made precise in Theorem 3 below.

To prove the stability estimate, we will be using the following Polarized Identity and the following Discrete Trace-Inverse Inequality:

- Polarized identity:
(45) $$2(a-c,b)=a^{2}-c^{2}-(a-b{)}^{2}+(b-c{)}^{2},\;\forall a,b,c\in R.$$
- The discrete trace-inverse inequality:
(46) DuhnΓˆ2≪CTIh-1DuhΩˆf'2
  where $C_{TI}$ is a positive constant, uniformly bounded from above with respect to the mesh characteristic size h for a family of shape-regular and quasi-uniform meshes, such as our domains defined in Section 3.1 [37].

To analyze the stability of the proposed method, it is convenient to rewrite the linear extrapolations described in Step 2 as a set of forward Euler problems [35]. In particular, Step 2 is equivalent to the following problem:

Step 2’: Given $u_{f,h}^{n+\theta }$, $w^{n}$, ${\dot{\eta }}_{h}^{n+\theta }$, $\eta_{h}^{n+\theta }$, $p_{p,h}^{n+\theta }$, compute $u_{f,h}^{n+1}$, ${\dot{\eta }}_{h}^{n+1}$, $u_{p,h}^{n+1}$, $p_{p,h}^{n+1}$, such that

(47) $$\begin{array}{l} \rho_{f}\int_{\Omega_{f}^{n}}\;\frac{u_{f,h}^{n+1}-u_{f,h}^{n+\theta }}{(1-\theta )\Delta t}\cdot \phi_{f,h}dx+\rho_{f}\int_{\Omega_{f}^{n}}\;\left(\left(u_{f,h}^{n}-w^{n}\right)\cdot \nabla \right)u_{f,h}^{n+\theta }\cdot \phi_{f,h}dx\\ \;-\int_{\Omega_{f}^{n}}\;p_{f,h}^{n+\theta }\nabla \cdot \phi_{f,h}dx+2\mu_{f}\int_{\Omega_{f}^{n}}\;D\left(u_{f,h}^{n+\theta }\right):D\left(\phi_{f,h}\right)dx\\ \;+\rho_{p}\int_{{\overset{ˆ}{\Omega }}_{p}}\;\frac{{\dot{\eta }}_{h}^{n+1}-{\dot{\eta }}_{h}^{n+\theta }}{(1-\theta )\Delta t}\cdot \xi_{p,h}dx+2\mu_{s}\int_{{\overset{ˆ}{\Omega }}_{p}}\;D\left(\eta_{h}^{n+\theta }\right)\;:D\left(\xi_{p,h}\right)dx\\ \;+\lambda_{p}\int_{{\overset{ˆ}{\Omega }}_{p}}\;\left(\nabla \cdot \eta_{h}^{n+\theta }\right)\left(\nabla \cdot \xi_{p,h}\right)dx-\alpha \int_{{\overset{ˆ}{\Omega }}_{p}}\;p_{p,h}^{n+\theta }\left(\nabla \cdot \xi_{p,h}\right)dx\\ \;+c_{0}\int_{{\overset{ˆ}{\Omega }}_{p}}\;\frac{p_{p,h}^{n+1}-p_{p,h}^{n+\theta }}{(1-\theta )\Delta t}\psi_{p,h}dx+\alpha \int_{{\overset{ˆ}{\Omega }}_{p}}\;\left(\nabla \cdot {\dot{\eta }}_{h}^{n+\theta }\right)\psi_{p,h}dx+\int_{{\overset{ˆ}{\Omega }}_{p}}\;\left(\nabla \cdot u_{p,h}^{n+\theta }\right)\psi_{p,h}dx\\ \;+\int_{{\overset{ˆ}{\Omega }}_{p}}\;{\left(\kappa^{n}\right)}^{-1}u_{p,h}^{n+\theta }\cdot \phi_{p,h}dx-\int_{{\overset{ˆ}{\Omega }}_{p}}\;p_{p,h}^{n+\theta }\left(\nabla \cdot \phi_{p,h}\right)dx-I_{h}^{*,n+\theta }\\ \;=\int_{\Gamma_{in}^{f}}\;P_{in}^{n+\theta }\left(\phi_{f,h}\cdot n\right)dS-\int_{\Gamma_{\mathrm{out}}^{f}}\;P_{\mathrm{out}}^{n+\theta }\left(\phi_{f,h}\cdot n\right)dS. \end{array}$$

The forward Euler version of Step 2, taking into account the assumptions used in stability analysis, is used in the following result.

#### Theorem 3.

*Let* $C_{TI}$ *be the constant from the discrete trace-inverse inequality* (46), *and* ς∈{-1,0,1}. *Furthermore, let the Nitsche’s penalty parameter* $\gamma_{f}>0$ *be such that*

(48) $$\gamma_{f}>(1+\varsigma ){\left(\epsilon_{f}\right)}^{-1},\;\text{where}\;\epsilon_{f}<\frac{1}{(1+\varsigma )C_{TI}}.$$

Then the following stability estimate holds:

(49) $$\begin{array}{l} \frac{\rho_{f}}{2}{\left\|u_{f,h}^{N}\right\|}_{{\overset{ˆ}{\Omega }}_{f}}^{2}+\frac{\rho_{p}}{2}{\left\|{\dot{\eta }}_{h}^{n+1}\right\|}_{{\overset{ˆ}{\Omega }}_{p}}^{2}+\frac{c_{0}}{2}{\left\|p_{p,h}^{n+1}\right\|}_{{\overset{ˆ}{\Omega }}_{p}}^{2}+\mu_{p}{\left\|D\left(\eta_{h}^{N}\right)\right\|}_{{\overset{ˆ}{\Omega }}_{p}}^{2}+\frac{\lambda_{p}}{2}{\left\|\nabla \cdot \eta_{h}^{N}\right\|}_{{\overset{ˆ}{\Omega }}_{p}}^{2}\\ \;+(2\theta -1)\Delta t\sum_{n=0}^{N-1}\;{\left\|u_{f,h}^{n+1}-u_{f,h}^{n}\right\|}_{{\overset{ˆ}{\Omega }}_{f}}^{2}+(2\theta -1)\Delta t\sum_{n=0}^{N-1}\;{\left\|{\dot{\eta }}_{h}^{n+1}-{\dot{\eta }}_{h}^{n}\right\|}_{{\overset{ˆ}{\Omega }}_{p}}^{2}\\ \;+(2\theta -1)\Delta t\sum_{n=0}^{N-1}\;{\left\|\eta_{h}^{n+1}-\eta_{h}^{n}\right\|}_{{\overset{ˆ}{\Omega }}_{p}}^{2}+(2\theta -1)\Delta t\sum_{n=0}^{N-1}\;{\left\|p_{p,h}^{n+1}-p_{p,h}^{n}\right\|}_{{\overset{ˆ}{\Omega }}_{p}}^{2}\\ \;+\mu_{f}\left(1-(1+\varsigma )\epsilon_{f}C_{TI}\right)\Delta t\sum_{n=0}^{N-1}\;{\left\|D\left(u_{f,h}^{n+\theta }\right)\right\|}_{{\overset{ˆ}{\Omega }}_{f}}^{2}\\ \;+\kappa^{-1}\Delta t\sum_{n=0}^{N-1}\;{\left\|u_{p,h}^{n+\theta }\right\|}_{{\overset{ˆ}{\Omega }}_{p}}^{2}+\beta \Delta t\sum_{n=0}^{N-1}\;{\left\|\left(u_{f,h}^{n+\theta }-{\dot{\eta }}_{h}^{n+\theta }\right)\cdot t\right\|}_{\overset{ˆ}{\Gamma }}^{2}\\ \;+\mu_{f}h^{-1}\Delta t\left(\gamma_{f}-(1+\varsigma )\epsilon_{f}^{-1}\right)\sum_{n=0}^{N-1}\;{\left\|\left(u_{f,h}^{n+\theta }-u_{p,h}^{n+\theta }-{\dot{\eta }}_{h}^{n+\theta }\right)\cdot n\right\|}_{\overset{ˆ}{\Gamma }}^{2}\\ \;\leq \frac{C_{T}^{2}C_{P}C_{K}^{2}}{2\mu_{f}}{\left\|P_{in}^{n+\theta }\right\|}_{\Gamma_{in}^{f}}^{2}+\frac{C_{T}^{2}C_{P}C_{K}^{2}}{2\mu_{f}}{\left\|P_{\mathrm{out}}^{n+\theta }\right\|}_{\Gamma_{\mathrm{out}}^{f}}^{2}. \end{array}$$

##### Proof.

To obtain this stability estimate, we start by replacing the test functions in (40) and (41) by the solutions: $\phi_{f,h}=u_{f,h}^{n+\theta }$, $\psi_{f,h}=p_{f,h}^{n+\theta }$, $\phi_{p,h}=u_{p,h}^{n+\theta }$, $\psi_{p,h}=p_{p,h}^{n+\theta }$, $\xi_{p,h}={\dot{\eta }}_{h}^{n+\theta }$ to obtain:

(50) $$\begin{array}{l} \frac{\rho_{f}}{2\theta \Delta t}\left({\left\|u_{f,h}^{n+\theta }\right\|}_{{\overset{ˆ}{\Omega }}_{f}}^{2}+{\left\|u_{f,h}^{n+\theta }-u_{f,h}^{n}\right\|}_{{\overset{ˆ}{\Omega }}_{f}}^{2}-{\left\|u_{f,h}^{n}\right\|}_{{\overset{ˆ}{\Omega }}_{f}}^{2}\right)+2\mu_{f}{\left\|D\left(u_{f,h}^{n+\theta }\right)\right\|}_{{\overset{ˆ}{\Omega }}_{f}}^{2}\\ \;+\frac{\rho_{p}}{2\theta \Delta t}\left({\left\|{\dot{\eta }}_{h}^{n+\theta }\right\|}_{{\overset{ˆ}{\Omega }}_{p}}^{2}+{\left\|{\dot{\eta }}_{h}^{n+\theta }-{\dot{\eta }}_{h}^{n}\right\|}_{{\overset{ˆ}{\Omega }}_{p}}^{2}-{\left\|{\dot{\eta }}_{h}^{n}\right\|}_{{\overset{ˆ}{\Omega }}_{p}}^{2}\right)\\ \;+\frac{c_{0}}{2\theta \Delta t}\left({\left\|p_{p,h}^{n+\theta }\right\|}_{{\overset{ˆ}{\Omega }}_{p}}^{2}+{\left\|p_{p,h}^{n+\theta }-p_{p,h}^{n}\right\|}_{{\overset{ˆ}{\Omega }}_{p}}^{2}-{\left\|p_{p,h}^{n}\right\|}_{{\overset{ˆ}{\Omega }}_{p}}^{2}\right)+\kappa^{-1}{\left\|u_{p,h}^{n+\theta }\right\|}_{{\overset{ˆ}{\Omega }}_{p}}^{2}\\ \;+\frac{\mu_{p}}{\theta \Delta t}\left({\left\|D\left(\eta_{h}^{n+\theta }\right)\right\|}_{{\overset{ˆ}{\Omega }}_{p}}^{2}-{\left\|D\left(\eta_{h}^{n}\right)\right\|}_{{\overset{ˆ}{\Omega }}_{p}}^{2}+{\left\|D\left(\eta_{h}^{n+\theta }\right)-D\left(\eta_{h}^{n}\right)\right\|}_{{\overset{ˆ}{\Omega }}_{p}}^{2}\right)\\ \;+\frac{\lambda_{p}}{2\theta \Delta t}\left({\left\|\nabla \cdot \eta_{h}^{n+\theta }\right\|}_{{\overset{ˆ}{\Omega }}_{p}}^{2}-{\left\|\nabla \cdot \eta_{h}^{n}\right\|}_{{\overset{ˆ}{\Omega }}_{p}}^{2}+{\left\|\nabla \cdot \eta_{h}^{n+\theta }-\nabla \cdot \eta_{h}^{n}\right\|}_{{\overset{ˆ}{\Omega }}_{p}}^{2}\right)\\ \;+\beta {\left\|\left(u_{f,h}^{n+\theta }-{\dot{\eta }}_{h}^{n+\theta }\right)\cdot t\right\|}_{\overset{ˆ}{\Gamma }}^{2}+\gamma_{f}\mu_{f}h^{-1}{\left\|\left(u_{f,h}^{n+\theta }-u_{p,h}^{n+\theta }-{\dot{\eta }}_{h}^{n+\theta }\right)\cdot n\right\|}_{\overset{ˆ}{\Gamma }}^{2}-𝒯\\ \;=\int_{\Gamma_{in}^{f}}\;P_{in}^{n+\theta }u_{f,h}^{n+\theta }\cdot ndS-\int_{\Gamma_{\mathrm{out}\;}^{f}}\;P_{\mathrm{out}\;}^{n+\theta }u_{f,h}^{n+\theta }\cdot ndS, \end{array}$$

where

(51) $$\begin{array}{l} 𝒯=\;\int_{\overset{ˆ}{\Gamma }}\;n\cdot \sigma_{f}\left(u_{f,h}^{n+\theta },p_{f,h}^{n+\theta }\right)n\left(u_{f,h}^{n+\theta }-u_{p,h}^{n+\theta }-{\dot{\eta }}_{h}^{n+\theta }\right)\cdot ndS\\ \;+\int_{\overset{ˆ}{\Gamma }}\;n\cdot \sigma_{f}\left(su_{f,h}^{n+\theta },-p_{f,h}^{n+\theta }\right)n\left(u_{f,h}^{n+\theta }-u_{p,h}^{n+\theta }-{\dot{\eta }}_{h}^{n+\theta }\right)\cdot ndS. \end{array}$$

Similarly, we replace the test functions in (47) by $\phi_{f,h}=u_{f,h}^{n+\theta }$, $\psi_{f,h}=p_{f,h}^{n+\theta }$, $\phi_{p,h}=u_{p,h}^{n+\theta }$, $\psi_{p,h}=p_{p,h}^{n+\theta }$, $\xi_{p,h}={\dot{\eta }}_{h}^{n+\theta }$. We use the polarized identity (45) and obtain the following equality:

(52) $$\begin{array}{l} \frac{\rho_{f}}{2(1-\theta )\Delta t}\left({\left\|u_{f,h}^{n+1}\right\|}_{{\overset{ˆ}{\Omega }}_{f}}^{2}-{\left\|u_{f,h}^{n+1}-u_{f,h}^{n+\theta }\right\|}_{{\overset{ˆ}{\Omega }}_{f}}^{2}-{\left\|u_{f,h}^{n+\theta }\right\|}_{{\overset{ˆ}{\Omega }}_{f}}^{2}\right)+2\mu_{f}{\left\|D\left(u_{f,h}^{n+\theta }\right)\right\|}_{{\overset{ˆ}{\Omega }}_{f}}^{2}\\ \;+\frac{\rho_{p}}{2(1-\theta )\Delta t}\left({\left\|{\dot{\eta }}_{h}^{n+1}\right\|}_{{\overset{ˆ}{\Omega }}_{p}}^{2}-{\left\|{\dot{\eta }}_{h}^{n+1}-{\dot{\eta }}_{h}^{n+\theta }\right\|}_{{\overset{ˆ}{\Omega }}_{p}}^{2}-{\left\|{\dot{\eta }}_{h}^{n+\theta }\right\|}_{{\overset{ˆ}{\Omega }}_{p}}^{2}\right)\\ \;+\frac{c_{0}}{2(1-\theta )\Delta t}\left({\left\|p_{p,h}^{n+1}\right\|}_{{\overset{ˆ}{\Omega }}_{p}}^{2}-{\left\|p_{p,h}^{n+1}-p_{p,h}^{n+\theta }\right\|}_{{\overset{ˆ}{\Omega }}_{p}}^{2}-{\left\|p_{p,h}^{n+\theta }\right\|}_{{\overset{ˆ}{\Omega }}_{p}}^{2}\right)+\kappa^{-1}{\left\|u_{p,h}^{n+\theta }\right\|}_{{\overset{ˆ}{\Omega }}_{p}}^{2}\\ \;+\frac{\mu_{p}}{(1-\theta )\Delta t}\left({\left\|D\left(\eta_{h}^{n+1}\right)\right\|}_{{\overset{ˆ}{\Omega }}_{p}}^{2}-{\left\|D\left(\eta_{h}^{n+\theta }\right)\right\|}_{{\overset{ˆ}{\Omega }}_{p}}^{2}-{\left\|D\left(\eta_{h}^{n+1}\right)-D\left(\eta_{h}^{n+\theta }\right)\right\|}_{{\overset{ˆ}{\Omega }}_{p}}^{2}\right)\\ \;+\frac{\lambda_{p}}{2(1-\theta )\Delta t}\left({\left\|\nabla \cdot \eta_{h}^{n+1}\right\|}_{{\overset{ˆ}{\Omega }}_{p}}^{2}-{\left\|\nabla \cdot \eta_{h}^{n+\theta }\right\|}_{{\overset{ˆ}{\Omega }}_{p}}^{2}-{\left\|\nabla \cdot \eta_{h}^{n+1}-\nabla \cdot \eta_{h}^{n+\theta }\right\|}_{{\overset{ˆ}{\Omega }}_{p}}^{2}\right)\\ \;+\beta {\left\|\left(u_{f,h}^{n+\theta }-{\dot{\eta }}_{h}^{n+\theta }\right)\cdot t\right\|}_{\overset{ˆ}{\Gamma }}^{2}+\gamma_{f}\mu_{f}h^{-1}{\left\|\left(u_{f,h}^{n+\theta }-u_{p,h}^{n+\theta }-{\dot{\eta }}_{h}^{n+\theta }\right)\cdot n\right\|}_{\overset{ˆ}{\Gamma }}^{2}-𝒯\\ \;=\int_{\Gamma_{in}^{f}}\;P_{in}^{n+\theta }u_{f,h}^{n+\theta }\cdot ndS-\int_{\Gamma_{\mathrm{out}}^{f}}\;P_{\mathrm{out}}^{n+\theta }u_{f,h}^{n+\theta }\cdot ndS. \end{array}$$

Multiplying (50) by θ and (52) by 1-θ, and adding the equations together, we get:

(53) $$\begin{array}{l} \frac{\rho_{f}}{2\Delta t}\left({\left\|u_{f,h}^{n+1}\right\|}_{{\overset{ˆ}{\Omega }}_{f}}^{2}-{\left\|u_{f,h}^{n+1}-u_{f,h}^{n+\theta }\right\|}_{{\overset{ˆ}{\Omega }}_{f}}^{2}+{\left\|u_{f,h}^{n+\theta }-u_{f,h}^{n}\right\|}_{{\overset{ˆ}{\Omega }}_{f}}^{2}-{\left\|u_{f,h}^{n}\right\|}_{{\overset{ˆ}{\Omega }}_{f}}^{2}\right)\\ \;+2\mu_{f}{\left\|D\left(u_{f,h}^{n+\theta }\right)\right\|}_{{\overset{ˆ}{\Omega }}_{f}}^{2}\\ \;+\frac{\rho_{p}}{2\Delta t}\left({\left\|{\dot{\eta }}_{h}^{n+1}\right\|}_{{\overset{ˆ}{\Omega }}_{p}}^{2}-{\left\|{\dot{\eta }}_{h}^{n+1}-{\dot{\eta }}_{h}^{n+\theta }\right\|}_{{\overset{ˆ}{\Omega }}_{p}}^{2}+{\left\|{\dot{\eta }}_{h}^{n+\theta }-{\dot{\eta }}_{h}^{n}\right\|}_{{\overset{ˆ}{\Omega }}_{p}}^{2}-{\left\|{\dot{\eta }}_{h}^{n}\right\|}_{{\overset{ˆ}{\Omega }}_{p}}^{2}\right)\\ \;+\frac{c_{0}}{2\Delta t}\left({\left\|p_{p,h}^{n+1}\right\|}_{{\overset{ˆ}{\Omega }}_{p}}^{2}-{\left\|p_{p,h}^{n+1}-p_{p,h}^{n+\theta }\right\|}_{{\overset{ˆ}{\Omega }}_{p}}^{2}+{\left\|p_{p,h}^{n+\theta }-p_{p,h}^{n}\right\|}_{{\overset{ˆ}{\Omega }}_{p}}^{2}-{\left\|p_{p,h}^{n}\right\|}_{{\overset{ˆ}{\Omega }}_{p}}^{2}\right)+\kappa^{-1}{\left\|u_{p,h}^{n+\theta }\right\|}_{{\overset{ˆ}{\Omega }}_{p}}^{2}\\ \;+\frac{\mu_{p}}{\Delta t}\left({\left\|D\left(\eta_{h}^{n+1}\right)\right\|}_{{\overset{ˆ}{\Omega }}_{p}}^{2}-{\left\|D\left(\eta_{h}^{n+1}\right)-D\left(\eta_{h}^{n+\theta }\right)\right\|}_{{\overset{ˆ}{\Omega }}_{p}}^{2}+{\left\|D\left(\eta_{h}^{n+\theta }\right)-D\left(\eta_{h}^{n}\right)\right\|}_{{\overset{ˆ}{\Omega }}_{p}}^{2}-{\left\|D\left(\eta_{h}^{n}\right)\right\|}_{{\overset{ˆ}{\Omega }}_{p}}^{2}\right)\\ \;+\frac{\lambda_{p}}{2\Delta t}\left({\left\|\nabla \cdot \eta_{h}^{n+1}\right\|}_{{\overset{ˆ}{\Omega }}_{p}}^{2}-{\left\|\nabla \cdot \eta_{h}^{n+1}-\nabla \cdot \eta_{h}^{n+\theta }\right\|}_{{\overset{ˆ}{\Omega }}_{p}}^{2}+{\left\|\nabla \cdot \eta_{h}^{n+\theta }-\nabla \cdot \eta_{h}^{n}\right\|}_{{\overset{ˆ}{\Omega }}_{p}}^{2}-{\left\|\nabla \cdot \eta_{h}^{n}\right\|}_{{\overset{ˆ}{\Omega }}_{p}}^{2}\right)\\ \;+\beta {\left\|\left(u_{f,h}^{n+\theta }-{\dot{\eta }}_{h}^{n+\theta }\right)\cdot t\right\|}_{\overset{ˆ}{\Gamma }}^{2}+\gamma_{f}\mu_{f}h^{-1}{\left\|\left(u_{f,h}^{n+\theta }-u_{p,h}^{n+\theta }-{\dot{\eta }}_{h}^{n+\theta }\right)\cdot n\right\|}_{\overset{ˆ}{\Gamma }}^{2}-𝒯\\ \;=\int_{\Gamma_{in}^{f}}P_{in}^{n+\theta }u_{f,h}^{n+\theta }\cdot ndS-\int_{\Gamma_{\mathrm{out}\;}^{f}}\;P_{\mathrm{out}\;}^{n+\theta }u_{f,h}^{n+\theta }\cdot ndS. \end{array}$$

Taking into account the extrapolations defined in Step 2, we have

$$\begin{array}{l} {\left\|u_{f,h}^{n+\theta }-u_{f,h}^{n}\right\|}_{{\overset{ˆ}{\Omega }}_{f}}^{2}-{\left\|u_{f,h}^{n+1}-u_{f,h}^{n+\theta }\right\|}_{{\overset{ˆ}{\Omega }}_{f}}^{2}=(2\theta -1){\left\|u_{f,h}^{n+1}-u_{f,h}^{n}\right\|}_{{\overset{ˆ}{\Omega }}_{f}}^{2},\\ {\left\|{\dot{\eta }}_{h}^{n+\theta }-{\dot{\eta }}_{h}^{n}\right\|}_{{\overset{ˆ}{\Omega }}_{p}}^{2}-{\left\|{\dot{\eta }}_{h}^{n+1}-{\dot{\eta }}_{h}^{n+\theta }\right\|}_{{\overset{ˆ}{\Omega }}_{p}}^{2}=(2\theta -1){\left\|{\dot{\eta }}_{h}^{n+1}-{\dot{\eta }}_{h}^{n}\right\|}_{{\overset{ˆ}{\Omega }}_{p}}^{2}\\ {\left\|\eta_{h}^{n+\theta }-\eta_{h}^{n}\right\|}_{{\overset{ˆ}{\Omega }}_{p}}^{2}-{\left\|\eta_{h}^{n+1}-\eta_{h}^{n+\theta }\right\|}_{{\overset{ˆ}{\Omega }}_{p}}^{2}=(2\theta -1){\left\|\eta_{h}^{n+1}-\eta_{h}^{n}\right\|}_{{\overset{ˆ}{\Omega }}_{p}}^{2}\\ {\left\|p_{p,h}^{n+\theta }-p_{p,h}^{n}\right\|}_{{\overset{ˆ}{\Omega }}_{p}}^{2}-{\left\|p_{p,h}^{n+1}-p_{p,h}^{n+\theta }\right\|}_{{\overset{ˆ}{\Omega }}_{p}}^{2}=(2\theta -1){\left\|p_{p,h}^{n+1}-p_{p,h}^{n}\right\|}_{{\overset{ˆ}{\Omega }}_{p}}^{2}. \end{array}$$

Using the Cauchy-Schwarz inequality and Young's inequality with $\epsilon_{f}>0$, we get:

$$\begin{array}{l} 𝒯\;\leq 2\mu_{f}(1+\varsigma ){\left\|D\left(u_{f,h}^{n+\theta }\right)n\right\|}_{\overset{ˆ}{\Gamma }}{\left\|\left(u_{f,h}^{n+\theta }-u_{p,h}^{n+\theta }-{\dot{\eta }}_{h}^{n+\theta }\right)\cdot n\right\|}_{\overset{ˆ}{\Gamma }}\\ \;\leq \mu_{f}(1+\varsigma )\epsilon_{f}h{\left\|D\left(u_{f,h}^{n+\theta }\right)\right\|}_{\overset{ˆ}{\Gamma }}^{2}+\mu_{f}(1+\varsigma ){\left(\epsilon_{f}h\right)}^{-1}{\left\|\left(u_{f,h}^{n+\theta }-u_{p,h}^{n+\theta }-{\dot{\eta }}_{h}^{n+\theta }\right)\cdot n\right\|}_{\overset{ˆ}{\Gamma }}^{2}. \end{array}$$

Applying the discrete trace-inverse inequality (46) we obtain:

$$\mu_{f}(1+\varsigma )\epsilon_{f}h{\left\|D\left(u_{f,h}^{n+\theta }\right)\right\|}_{\overset{ˆ}{\Gamma }}^{2}\leq \mu_{f}(1+\varsigma )\epsilon_{f}C_{TI}{\left\|D\left(u_{f,h}^{n+\theta }\right)\right\|}_{{\overset{ˆ}{\Omega }}_{f}}^{2}.$$

Therefore, the estimate of the boundary term is given by:

$$𝒯\leq \mu_{f}(1+\varsigma )\epsilon_{f}C_{TI}{\left\|D\left(u_{f,h}^{n+\theta }\right)\right\|}_{{\overset{ˆ}{\Omega }}_{f}}^{2}+\mu_{f}(1+\varsigma ){\left(\epsilon_{f}h\right)}^{-1}{\left\|\left(u_{f,h}^{n+\theta }-u_{p,h}^{n+\theta }-{\dot{\eta }}_{h}^{n+1}\right)\cdot n\right\|}_{\overset{ˆ}{\Gamma }}^{2}.$$

To estimate the right hand side of (53), we use the Cauchy-Schwarz, Young’s, Poincare’s and Korn’s inequalities to obtain:

∫ΓinfPinn+θuf,hn+θ⋅ndS+∫ΓoutfPoutn+θuf,hn+θ⋅ndS≤Pinn+θΓinf2uf,hn+θΓinf2+Poutn+θΓoutf2uf,hn+θΓoutf2≤CT2CPCK22μfPinn+θΓinf2+CT2CPCK22μfPoutn+θΓoutf2+μfDuf,hn+θΩˆf'2

where $C_{T}$, $C_{P}$ and $C_{K}$ are positive constants. Combining the estimates above with (53), multiplying the results by Δt and calculating the sum from n=0 to N-1, we obtain estimate (49). □

Therefore, we have proved (for the fixed fluid domain case) that our proposed numerical scheme (40)–(42) is unconditionally stable.

### 3.2. Discretization of the Coupled Advection-Reaction-Diffusion Problem

As in the case of the FSI problem above, we use a Finite Element Method-based approach to solve the coupled advection–reaction–diffusion problem (28), (29) and (32). To specify the discretized problem, we must first state the weak formulation of (28), (29) and (32).

#### Weak formulation.

Let us assume that the fluid–structure interface Γ(t) is Lipschitz. Furthermore, let $u_{f}\in V^{f}$, and let ${\tilde{u}}_{p}\in L^{2}\left(0,T;L^{2}\left(\Omega_{p}(t)\right)\right)$ be such that $\nabla \cdot u_{p}\in L^{2}\left(0,T;L^{2}\left(\Omega_{p}(t)\right)\right)$. Assume that the domain velocities $w_{f}$ and $w_{p}$ are such that $w_{f}\in L^{\infty }\left(0,T;W^{1,\infty }\left(\Omega_{f}(t)\right)\right)$ with $\nabla \cdot w_{f}\in L^{\infty }\left(0,T;W^{1,\infty }\left(\Omega_{f}(t)\right)\right)$, and $w_{p}\in L^{\infty }(0,T;\left.L^{2}\left(\Omega_{p}(t)\right)\right)\cap L^{2}\left(0,T;H^{1}\left(\Omega_{p}(t)\right)\right)$. We introduce the following solution space for oxygen concentrations $C_{f}$ in the fluid channel and $C_{p}$ in the hydrogel:

$$\begin{array}{c} 𝒞=\left\{\left(C_{f},C_{p}\right)\in L^{2}\left(0,T;H^{1}\left(\Omega_{f}(t)\right)\right)\times L^{2}\left(0,T;H^{1}\left(\Omega_{p}(t)\right)\right)|\right.\\ \left.\partial_{t}C_{f}\in L^{2}\left(0,T;{\left(H^{1}\left(\Omega_{f}(t)\right)\right)}^{*}\right),\partial_{t}C_{p}\in L^{2}\left(0,T;{\left(H^{1}\left(\Omega_{p}(t)\right)\right)}^{*}\right),C_{f}=C_{p}\;\mathrm{on}\;\Gamma (t)\right\}. \end{array}$$

##### Definition 1.

*A function* $\left(C_{f},C_{p}\right)\in 𝒞$ *is said to be a weak solution of the coupled advection–reaction–diffusion problem* (28), (29) *and* (32) *if* ∀(φ,ψ)∈𝒞,

(54) $$\begin{array}{l} \;\int_{0}^{T}\;\int_{\Omega_{f}(t)}\;\left\{{\left.\partial_{t}C_{f}\right|}_{x_{0}}\varphi -\nabla \varphi \cdot \left[\left(u_{f}-w_{f}\right)C_{f}-D_{f}\nabla C_{f}\right]+\varphi \left(\nabla \cdot w_{f}\right)C_{f}\right\}dxdt\\ \;+\int_{0}^{T}\;\int_{\Omega_{p}(t)}\;\left\{{\left.\partial_{t}C_{p}\right|}_{x_{0}}\psi -\nabla \psi \cdot \left[\left({\tilde{u}}_{p}-w_{p}\right)C_{p}-D_{p}\nabla C_{p}\right]+\psi \left(\nabla \cdot w_{p}\right)C_{p}\right\}dxdt\\ \;=\int_{0}^{T}\;\int_{\Omega_{p}(t)}\;R_{max}\frac{C_{p}}{C_{p}+C_{MM}}\delta \left(C_{p}>C_{cr}\right)\psi dxdt. \end{array}$$

This weak formulation is derived by multiplying Equations (28) and (29) by the corresponding test functions, integrating by parts, and using the coupling conditions (32) at the fluid structure interface Γ(t).

##### Discretization.

We, again, assume that the shapes of $\Omega_{f}(t)$ and $\Omega_{p}(t)$ are polygons/tetrahedra, and denote by $T_{h}^{f}$ and $T_{h}^{p}$ the uniform conforming triangulations of $\Omega_{f}(t)$ and $\Omega_{p}(t)$, respectively. We define finite element space ${𝒞}_{h}\subset 𝒞$, and use the Backward Euler method to discretize the problem in time. The corresponding fully discretized coupled problem for oxygen concentration reads:

$$\begin{array}{l} \;\int_{\Omega_{f}^{n}}\;\left\{{\left.\frac{C_{f,h}^{n+1}-C_{f,h}^{n}}{\Delta t}\right|}_{x_{0}}\varphi_{h}-\nabla \varphi_{h}\cdot \left[\left(u_{f,h}^{n}-w_{f,h}^{n}\right)C_{f,h}^{n+1}-D_{f}\nabla C_{f,h}^{n+1}\right]+\varphi_{h}\left(\nabla \cdot w_{f,h}^{n}\right)C_{f,h}^{n+1}\right\}dx\\ \;+\int_{\Omega_{p}^{n}}\;\left\{{\left.\frac{C_{p,h}^{n+1}-C_{p,h}^{n}}{\Delta t}\right|}_{x_{0}}\psi_{h}-\nabla \psi_{h}\cdot \left[\left({\tilde{u}}_{p,h}^{n}-w_{p,h}^{n}\right)C_{p,h}^{n+1}-D_{p}\nabla C_{p,h}^{n+1}\right]+\psi_{h}\left(\nabla \cdot w_{p,h}^{n}\right)C_{p,h}^{n+1}\right\}dx\\ \;=\int_{\Omega_{p}^{n}}\;\left(R_{max}\frac{C_{p,h}^{n}}{C_{p,h}^{n}+C_{MM}}\delta \left(C_{p,h}^{n}>C_{cr}\right)\right)\psi_{h}dx, \end{array}$$

where $\left({𝒞}_{f,h}^{n+1},C_{p,h}^{n+1}\right)\in {𝒞}_{h}$, and $\left(\varphi_{h},\psi_{h}\right)\in {𝒞}_{h}$.

### 3.3. Parameter Estimation Using Encoder–Decoder Convolution Neural Networks and Smoothed Particle Hydrodynamics

Local information about hydraulic permeability of the 3D islet chamber is crucial for correctly approximating oxygen supply to the pancreatic islets seeded in the chamber containing a gel poroelastic matrix. While **global** hydraulic permeability can be estimated using experiments, the **local** properties are difficult to obtain experimentally. This is why we use our in-house Smoothed Particle Hydrodynamics (SPH) solver, developed based on the works [38–40], and experimentally validated in [41], to estimate the values of equilibrium permeability $\kappa_{0}$ from (5) at every point (x,y,z) in the poroelastic hydrogel. To achieve equilibrium permeability, constant pressure drop is prescribed at the inlet and outlet.

The SPH is a weighted interpolation method which represents all bulk properties of the fluid at a certain location in space with a discrete interpolation over a set of surrounding particles [38–41]. The particles corresponding to the hydrogel matrix are fixed in a random fashion over the hydrogel domain, satisfying a certain porosity volume fraction condition, while the moving particles describing the fluid satisfy a system of differential equations corresponding to the Navier–Stokes/Stokes equations. The ratio between the fixed particles and moving particles is given a priori, and it corresponds to the porosity of the hydrogel. At the inlet and outlet of the domain, the particles are leaving and entering the domain so that for every exiting particle, there is a new particle assigned at the inlet, with a given inlet velocity. A result of one such simulation is shown in Figure 8: we see a 2D slice of a 3D hydrogel where the inlet is on the left, and the outlet on the right. The first two panels in this figure show the magnitude of fluid/plasma filtration velocity and pressure, respectively. The last two panels on this figure show the permeability coefficients ${\left[\kappa_{0}\right]}_{11}$ and ${\left[\kappa_{0}\right]}_{33}$ obtained by locally applying Darcy law.

Since 3D SPH simulations are computationally expensive to be run for every single hydrogel geometry, we resort to the Encoder–Decoder Convolution Neural Networks (CNN), see, e.g., [42–44], to obtain the macro-scale permeability tensor, based on the microscale hydrogel geometry. We use synthetic data from the SPH simulations to train the network and obtain a macro-scale permeability tensor as a function of (x,y,z) for either synthetic/numerically generated hydrogels, or for the actual hydrogels whose geometry is obtained from imaging data.

The main idea behind the Encoder–Decoder Neural Network applied to our problem is based on treating the geometry of the gel poroelastic matrix as the input, and train the Encoder-decoder based CNN over a large set of synthetic data obtained using our pre-validated steady state simulations, to estimate the local hydraulic permeability tensor $\kappa_{0}$ as an output of the CNN. The main steps are as follows:

1. **Create an ensemble** of 100 poroelastic gel matrix geometries with different porosity by using SPH to distribute the solid particles in the hydrogel. The hydrogel is divided into boxes and treated as an image. Every box (cf. pixel) contains the information about the density of the non-moving SPH particles in that box (cf. pixel intensity in terms of image processing).
2. **Run SPH simulations** for each poroelastic matrix geometry to obtain the corresponding filtration flow and pressure, as illustrated in Figure 8a,b.
3. **Post-processing**: At each location in the chamber, compute the local hydraulic permeability tensor using data from step 2 above, see Figure 8c,d, and use it as training data (permeability map) for the Encoder–Decoder CNN.
4. **Train the Encoder–Decoder CNN** with the density data and corresponding permeability map obtained from steps 2 and 3. We use TensorFlow as our platform. The encoder contains several Convolution and Dense layers, and the decoder is just the reflection of those layers in the encoder.
5. **Feed** a new density matrix to the Encoder-decoder CNN and predict the local values of the hydraulic conductivity tensor for a new porous medium chamber.

While CNN training is an expensive part of this approach, it is performed “off-line” and only once. Once this is completed, getting new parameter values for different poroelastic matrix geometries is fast, and does not require the expensive SPH simulations. This approach is significantly “cheaper” computationally than generating and running new SPH simulations each time a new islet geometry is to be tested.

### 3.4. Parallel Implementation and Convergence Test

Our macro-scale solvers have been implemented within the FEniCS platform [45]. The macro-scale solvers, and the the nano-scale SPH solver, have both been **parallelized**. The nano-scale SPH solver has been running on a GPU node on the Savio cluster at UC Berkeley. Each GPU node is equipped with two Nvidia K80 GPU cards, where each card can run up to 2496 CUDA cores. One typical SPH simulation uses one whole node with a total of 2496 cores. The FSI solver has been running on the General Savio node pool. Each node is equipped with two Intel Xeon 12 core processors and 128 GB memory. We use 8 cores on each node, with 16 allocated nodes. We get nearly linear speedup for our solver implemented in FEniCS before reaching the memory limit for each node. FEniCS parallelization is different from the traditional approaches as it runs N identical copies of code in parallel, where N refers to the number of cores, and the original problem is divided into N subproblems. There is no master node to manage running the job, and each individual core executes its own job with gathering only the information it needs.

#### Convergence test of the FSI θ-scheme.

We conclude this section by showing that our proposed one legged ‘θ–like' method for the FSI problem, presented in Section 3.1, has higher order accuracy than the currently available Navier–Stokes-Biot schemes reported in [27,31]. In our convergence test, as stated above at the beginning of Section 2.1, we assume the thick poroelastic structure to be homogeneous and isotropic, and the stress tensor to be given by the first Piola-Kirchhoff stress tensor: $\Sigma (d)=2\mu_{s}\epsilon (d)+\lambda_{s}(\nabla \cdot d)I$, where $\epsilon (d)=\frac{\nabla d+(\nabla d{)}^{T}}{2}$ is the strain rate tensor, $\mu_{s}$ and $\lambda_{s}$ are the Lamé constants, which are related to Young’s modulus $E_{s}$ and the Poisson’s ratio $v_{s}$ via:

$$\mu_{s}=\frac{E_{s}}{2\;+\;2v_{s}},\;\lambda_{s}=\frac{E_{s}v_{s}}{\left(1\;+\;v_{s}\right)\left(1\;-\;2v_{s}\right)}.$$

We solve our FSI problem using the *method of manufactured solutions*, which were derived assuming that the fluid domain remains fixed during the simulation. The computational domain consists of a unit cube, where the top part of the cube corresponds to the fluid and the bottom part corresponds to the poroelastic structure, i.e., ${\overset{ˆ}{\Omega }}_{f}=(0,1)\times (0,1)\times (0,0.5)$ and ${\overset{ˆ}{\Omega }}_{p}=(0,1)\times (0,1)\times (-0.5,0)$. The exact solution is given by:

$$\begin{array}{l} \eta_{\mathrm{ref}\;}\;=\left[\begin{array}{c} sin(\pi t)(cos(y)-3x)\\ sin(\pi t)(y+1)\\ \frac{2}{3}sin(\pi t)z \end{array}\right],\\ p_{p,ref}\;=sin\left(\pi t+\frac{\pi }{4}\right)sin(\pi x)cos(0.5\pi y)-\frac{4}{3}\pi cos(\pi t),\\ u_{f,ref}\;=\left[\begin{array}{c} \pi cos(\pi t)(cos(y)-3x)\\ \pi cos(\pi t)(y+1)\\ \frac{2}{3}\pi cos(\pi t)z \end{array}\right],\;p_{f,ref}=sin\left(\pi t+\frac{\pi }{4}\right)sin(\pi x)cos(0.5\pi y). \end{array}$$

Forcing terms, $u_{p,ref}$ and ${\dot{\eta }}_{ref}$, as well as the boundary conditions, are computed using the exact solution. We note that the exact velocity is not divergence-free, in which case we also add a forcing term to the mass conservation equation:

$$\nabla \cdot u_{f}=F_{m},$$

where $F_{m}$ is computed using the exact solution. We impose the following boundary conditions

$$\begin{array}{ll} u_{f}=u_{f,ref}\; & \mathrm{on}\;\partial {\overset{ˆ}{\Omega }}_{p}\backslash (\overset{ˆ}{\Gamma }\cup {\overset{ˆ}{\Gamma }}^{*}),\\ \sigma_{f}\left(u_{f},p_{f}\right)n=\sigma_{f}\left(u_{f,ref},p_{f,ref}\right)n\; & \mathrm{on}\;{\overset{ˆ}{\Gamma }}^{*},\\ \eta_{f}=\eta_{f,ref}\; & \mathrm{on}\;\partial {\overset{ˆ}{\Omega }}_{p}\backslash {\overset{ˆ}{\Gamma }}^{'},\\ p_{p}=p_{p,ref}\; & \mathrm{on}\;\partial {\overset{ˆ}{\Omega }}_{p}\backslash \overset{ˆ}{\Gamma }, \end{array}$$

where ${\overset{ˆ}{\Gamma }}^{*}$ is the fluid external boundary corresponding to the plane x=0. The parameter values used in this simulation are all set to one: $\rho_{f}=\mu_{f}=\rho_{p}=\mu_{S}=\lambda_{S}=\alpha =c_{0}=\beta =1$ and κ is equal to the identity matrix. We use =0.5+Δt, $\gamma_{f}=10^{4}$, and the final time is set to T=0.2. In this case, a second-order convergence is expected.

Our convergence test was conducted on a fixed domain, where the mesh was refined together with the time step. In particular, the following temporal and spatial parameters are used:

$$(\Delta t,h)={\left\{\frac{4\cdot 10^{-2}}{2^{i}},\frac{0.5}{2^{i}}\right\}}_{i=0}^{2}.$$

We use $P_{2}-P_{1}$ elements for the fluid velocity and pressure, $P_{2}$ elements for the structure displacement, and $P_{2}-P_{1}$ elements for the filtration velocity and porous pressure. We compute the relative errors for $u_{f}$, $p_{f}$, $u_{p}$, $p_{p}$, η, ξ, defined by

$$\begin{array}{l} e_{k}=\frac{{\left\|u_{k}-u_{k,ref}\right\|}_{L^{2}\left({\overset{ˆ}{\Omega }}_{k}\right)}}{{\left\|u_{k,ref}\right\|}_{L^{2}\left({\overset{ˆ}{\Omega }}_{k}\right)}},\;e_{p,k}=\frac{{\left\|p_{k}-p_{k,ref}\right\|}_{L^{2}\left({\overset{ˆ}{\Omega }}_{k}\right)}}{{\left\|p_{k,ref}\right\|}_{L^{2}\left({\overset{ˆ}{\Omega }}_{k}\right)}},\;k=f,p,\\ e_{\eta }=\frac{{\left\|\eta -\eta_{ref}\right\|}_{S}}{{\left\|\eta_{ref}\right\|}_{S}},\;e_{\dot{\eta }}=\frac{{\left\|\dot{\eta }-{\dot{\eta }}_{ref}\right\|}_{L^{2}\left({\overset{ˆ}{\Omega }}_{p}\right)}}{{\left\|{\dot{\eta }}_{ref}\right\|}_{L^{2}\left({\overset{ˆ}{\Omega }}_{p}\right)}}, \end{array}$$

where $\|\cdot {\|}_{S}$ is the structure energy norm defined by

$$\|\eta {\|}_{S}^{2}=2\mu_{S}\|D(\eta ){\|}_{L^{2}\left({\overset{ˆ}{\Omega }}_{p}\right)}^{2}+\lambda_{S}\|\nabla \cdot \eta {\|}_{L^{2}\left({\overset{ˆ}{\Omega }}_{p}\right)}^{2}.$$

The results are reported in Tables 3 and 4.

This shows that our one legged ‘θ-like’ scheme is second-order accurate for the fluid velocity, structure displacement and velocity, and filtration pressure, and therefore has higher-order accuracy than other Navier–Stokes-Biot schemes reported in [27,31], which are only first-order accuracy in time.

## 4. Numerical Results

We study numerically the current prototype of the bioartificial pancreas, shown in Figures 4 and 5, and propose modifications in the design that promise to lead to increased filtration flow and increased oxygen concentration.

Tables 1 and 2 show the parameter values used in the simulations. The initial and boundary conditions are specified in Section 2.

### 4.1. One Outlet—Current Prototype Design

We start by considering the design shown in Figure 4. As mentioned above, in this design one outlet is associated with the outlet gasket. A detailed computational domain showing the outlet cylinder, the top gasket, the islet chamber with drilled ultrafiltrate channels, the bottom gasket and the four inlet nano-pore size membranes (the four squares at the bottom of the chamber) is shown in Figure 9 top.

Figure 9 bottom shows filtration flow streamlines with the colors denoting magnitude of filtration velocity. Red denotes high and blue low filtration velocity. As expected, the filtration velocity at the inlet and near the inlet at the bottom gasket is high.

The flow then enters the islet chamber both via the ultrafiltrate channels and also through the poroelastic hydrogel away from the channels. We see high velocity at the bottom of the islet chamber adjacent to the gasket-hydrogel interface, showing strong filtrate flow everywhere. As we move up further away from the bottom gasket, the filtrate flow is diminished, and most flow takes place through the ultrafiltrate channels. It is therefore expected that oxygen concentration would be greatest near the inlet to the islet chamber and close to the ultrafiltrate channels, rather than near the outlet of the chamber.

Indeed Figure 10 shows oxygen concentration at two different times $t_{1}$ and $t_{2}>t_{1}$. We see how oxygen populates the islet chamber, with red denoting high oxygen concentration, blue low oxygen concentration, and white intermediate oxygen concentration.

One can observe high oxygen concentration at the inlet membranes, in the gasket close to the islet chamber, and in much of the islet chamber, with highest oxygen concentration closer to the inlet. Furthermore, when comparing the left “half” of the chamber, which is closest to the outlet, we see that it has higher oxygen concentration than the right “half” of the chamber. We attribute this to the larger bulk flow closer to the outlet.

In fact, to investigate this situation further, we simulated the flow in the entire set up, as shown in Figure 11, with six semi-permeable nanopore membranes and flow through the ultrafiltrate channels, as shown in Figure 11 top. The top gasket collects all the outflow which leaves the device through one ultrafiltrate outlet.

As shown in Figure 11 top, the bulk of the flow occurs through the membranes/islet chambers closest to the outlet (the first three membranes closest to the outlet). Here red denotes high flow and blue low flow, as before. We conclude that adding several additional outlets to the top gasket would improve filtration flow everywhere within the islet chamber situated between the membranes and the top gasket. This is investigated next.

### 4.2. Two Outlets versus One Outlet

Here we investigate the influence of two outlets in the design shown in Figure 12 top, on filtration flow and oxygen concentration.

Figure 12 top shows the computational domain, and Figure 12 bottom shows the filtration velocity streamlines, with colors corresponding to the velocity magnitude. Two interesting observations can be made from the results in Figure 12:

- The presence of the second outlet improves the flow through the part of the islet chamber closest to that outlet (see Figure 12 right).
- The staggered distribution of islet chamber and membranes underneath the chamber, increases transverse flow through the islet chamber. This is shown by the angled streamlines in Figure 12 right.

Increased transverse flow through the islet chamber away from the ultrafiltrate channels can also be seen in Figure 13. Figure 13 shows a view from the top at two device designs: the one on the left has one outlet, the one on the right has two outlets. We can see an increase in transverse (diagonal) flow through the islet chamber, shown with red streamlines, in the design with two outlets. A combination of the presence of the second outlet and the “misalignment between the islet chamber and the two inlet membranes in the right half of the chamber is responsible for the increase in transverse (diagonal) flow.

The presence of the second outlet also helps avoid recirculation zones in the top gasket. Recirculation zones can be seen in Figure 13 left where the right-half of the top gasket shows areas of recirculation flow. This is even more pronounced in Figure 14 where a large recirculation zone in the top gasket away from the outlet can be seen.

We further investigated the influence of two outlets on oxygen concentration in the islet chamber. Figure 15 shows a comparison in oxygen concentration between the two designs.

The figure on the left corresponds to the design with one outlet, and the figure on the right corresponds to the design with two outlets. One can see that the design with two outlets shown on the right has larger regions with high oxygen concentration, as well as larger regions with dark red color indicating higher oxygen concentration.

To obtain more detailed information about oxygen concentration in the design with two outlets, we present a 2D slice through a 3D islet chamber, shown in Figures 16 and 17.

Figure 16 left shows the islet chamber with flow streamlines, with the flow entering the chamber from the top, and leaving at the bottom. On the right we see the corresponding oxygen concentration distributed within the chamber. Red is high, and dark blue low oxygen concentration, with the maximum concentration equal to 1.06 × 10^−7^ mol/cm^3^.

Figure 17 shows a 2D slice through the 3D islet chamber. We see, again, that high oxygen concentration occurs near the inlet, and close to the filtration channels (red drop-like vertical structures). The dark blue region where oxygen concentration is low corresponds to the area just under the frame of the membranes where there is no filtration flow entering the islet chamber. In both figures the flow comes from the top and leaves the islet chamber at the bottom.

### 4.3. Hydrogel Elasticity

Finally, we investigate the influence of hydrogel elasticity on flow and oxygen concentration. This is particularly important since elasticity can be controlled in the fabrication of agarose hydrogel scaffolds, fabricated using the approaches presented in [1]. Hydrogel elasticity is directly related to cells viability [46].

To investigate the influence of hydrogel elasticity on flow and oxygen concentration we performed two simulations: one assumes poroelasticity of a hydrogel, as described by the Biot model in Section 2.1, and the other assumes that hydrogels are rigid, and uses Darcy equation to calculate filtration flow through the hydrogel. We found that hydrogel elasticity improves both flitration flow and oxygen concentration within the hydrogel. Figure 18 shows the propagation of oxygen front (a level surface of oxygen concentration) as a function of time. The four panels in this figure show four snap shots of oxygen front propagation increasing in time from (a) to (d). Indeed, one can see that in the case of a poroelastic hydrogen, shown on the left in each of the four panels, oxygen front travels faster, and reaches the top gasket before the oxygen front in the rigid hydrogel, shown on the right in each of the four figures. Thus, elasticity of a hydrogel seems to be conducive to increased filtration flow, cf. Figure 19 below, and faster oxygenation of the islet chamber.

We further investigated the filtration velocity for the two cases, Biot versus Darcy, and found that indeed, there is a significant difference in filtration velocity between the two models, while the maximum velocity occurs for the Biot model and is equal to 3.5 cm/s, the maximum difference in the two velocities is 1 cm/s, which is almost one third of the maximal filtration velocity. We attribute this difference to the fluid pressure-related swelling of the hydrogel pores, accommodating more fluid, especially near the inlet into the hydrogel region. The difference between the filtration fluid velocity between the two scenarios is shown in Figure 19.

We conclude this section by comparing the overall oxygen concentration within three hydrogels with three different elastic moduli: alginate hydrogel with Youngs modulus $E=4\times 10^{4}$ Pascals, alginate with $E=0.75\times 10^{6}\;Pa$, and a very stiff scaffold made of Polycaprolactone (PCL) with $E=1.3\times 10^{8}\;Pa$. All the other properties, such as porosity and permeability, were assumed to be the same.

Figure 20 shows three curves of oxygen concentration over time, one for each cell scaffold. We see that there is a significant increase in oxygen concentration within the elastic alginate scaffolds with Youngs moduli $E=4\times 10^{4}\;Pa$ and $E=0.75\times 10^{6}$, in comparison with the stiff Polycaprolactone (PCL) scaffold with $E=1.3\times 10^{8}\;Pa$ modeled using Darcy model. Figure 20 shows that oxygen concentration in the most elastic scaffold, namely alginate gel with $E=4\times 10^{4}\;Pa$, is 30% higher than that in the stiffest scaffold considered in this study. This is particularly interesting considering that scaffold elasticity can be controlled during their fabrication, and that elastic scaffolds improve cell viability [46].

## 5. Conclusions

We developed a multi-scale mathematical and computational model to study cell encapsulation and design of an implantable bioartificial pancreas (iBAP). The macro-scale models include a fluid–structure interaction (FSI) model describing the flow of blood plasma through a poroelastic hydrogel, and a set of two coupled advection–reaction–diffusion models defined on moving domains: the hydrogel and two gaskets adjacent to the hydrogel. A novel second-order accurate finite element numerical scheme was designed to solve the FSI model. The scheme is based on a Cauchy’s θ-like method with Nitsche approach to impose the coupling conditions. We prove rigorously that the resulting scheme is unconditionally stable when Nitsche’s parameter is larger than a certain quantity, and we show that the method is second-order accurate in time using the method of manufactured solutions. At the micro-scale, Smoothed Particle Hydrodynamics (SPH) simulations are used to simulate local hydraulic permeability for a given hydrogel macro-architecture, from where hydrogel-specific macro-scale permeability tensor is derived. To avoid expensive 3D SPH simulations for each new hydrogel structure, Endoder-Decoder Neural Networks are used for parameter estimation of the macro-scale permeability tensor based on the micro-scale hydrogel architecture.

Our computational results show that:

1. Oxygen concentration and filtration flow through hydrogel scaffolds are significantly affected by the position and number of the ultrafiltrate outlets. The ultrafiltrate outlets should be (equi)distributed to uniformly cover the entire array of cell scaffolds.
2. Hydrogel elasticity significantly affects oxygen concentration and filtration flow through scaffolds. Highly elastic scaffolds have a higher capacity for oxygen transfer.
3. Oxygen concentration is largest near the flow inlet into the scaffold, and near the drilled ultrafiltrate channels.

The mathematical and computational approaches developed in this work provide a benchmark tool for computational analysis of not only iBAP, but also, more generally, of cell encapsulation strategies used in the design of devices for cell therapy and bio-artificial organs.

Extensions of this work include geometric optimization of ultrafiltrate channels’ distribution maximizing oxygen concentration within a given hydrogel, the development of different, more efficient, numerical methods to simulate the micro-scale ultrafiltrate flow, and inclusion of an advection–reaction–diffusion model for insulin concentration.

## Figures and Tables

**Figure 1.**
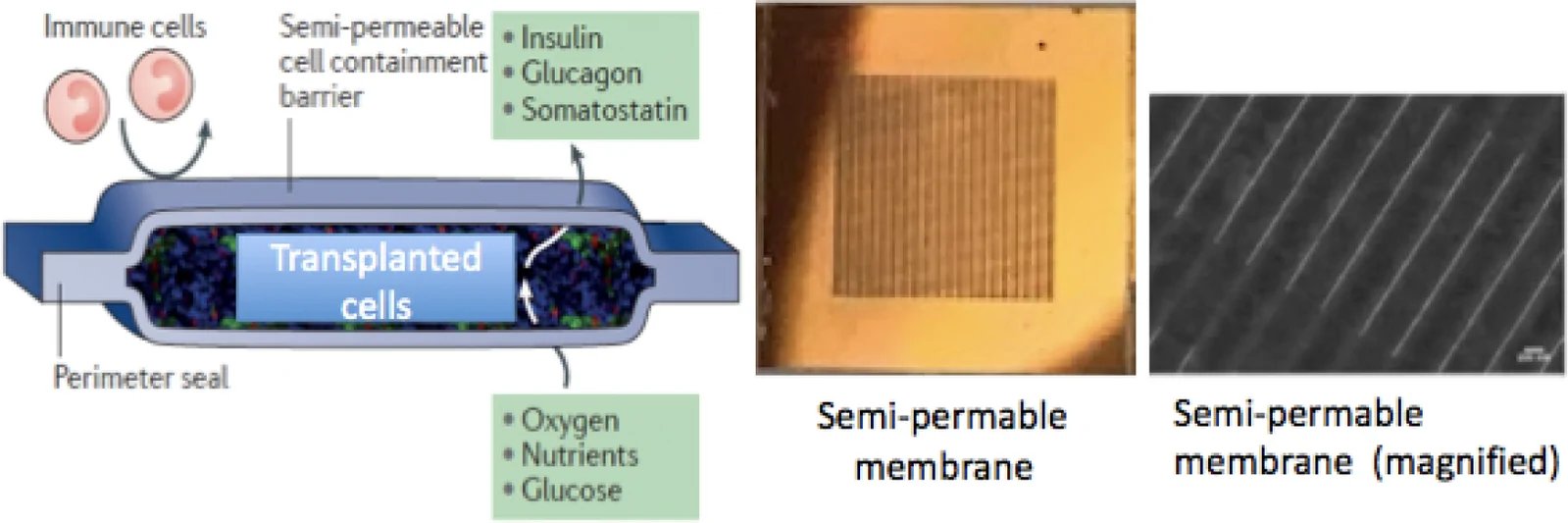
(**Left**): An illustration of an islet encapsulation device [6]; (**Middle**): Semi-permeable silicon membrane; (**Right**): A scanning electron microscope (SEM) image of the membrane surface illustrating slits 2 μm in length and 7 nm is width (courtesy of Dr. Roy).

**Figure 2.**
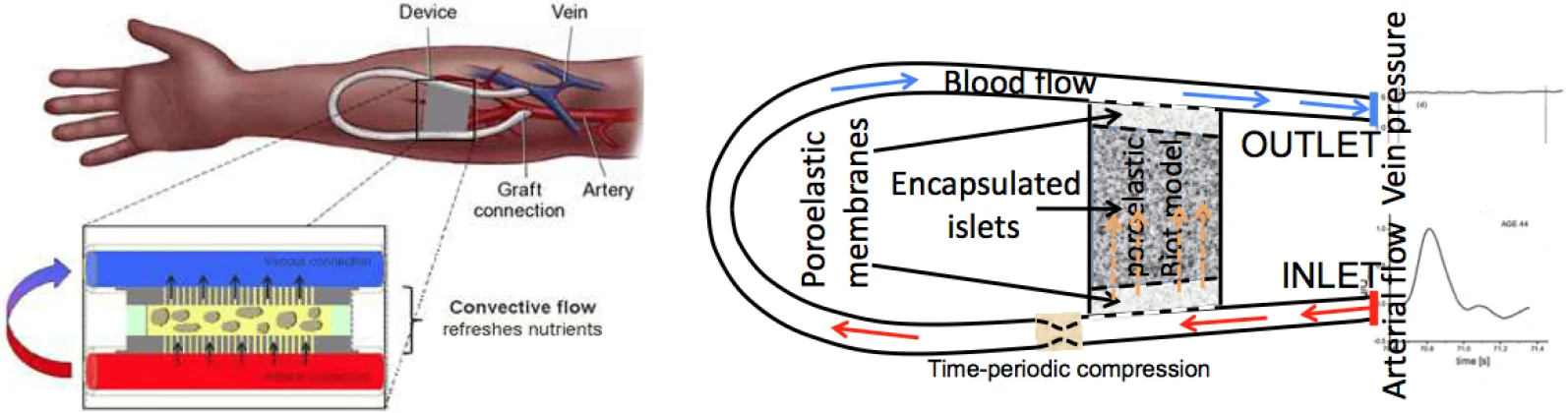
(**Left**): An illustration of the implantable intravascular bioartificial pancreas device in the arm of a T1D patient (from [5]). (**Right**): A sketch showing the graft and the encapsulation chamber consisting of two poroelastic membranes and the islet chamber in the middle. The figure also includes our suggestion for the placement of a time-periodic compression device downstream from the chamber, to increase convective flow into the chamber and flush out albumin deposits near the membrane observed in steady flow.

**Figure 3.**
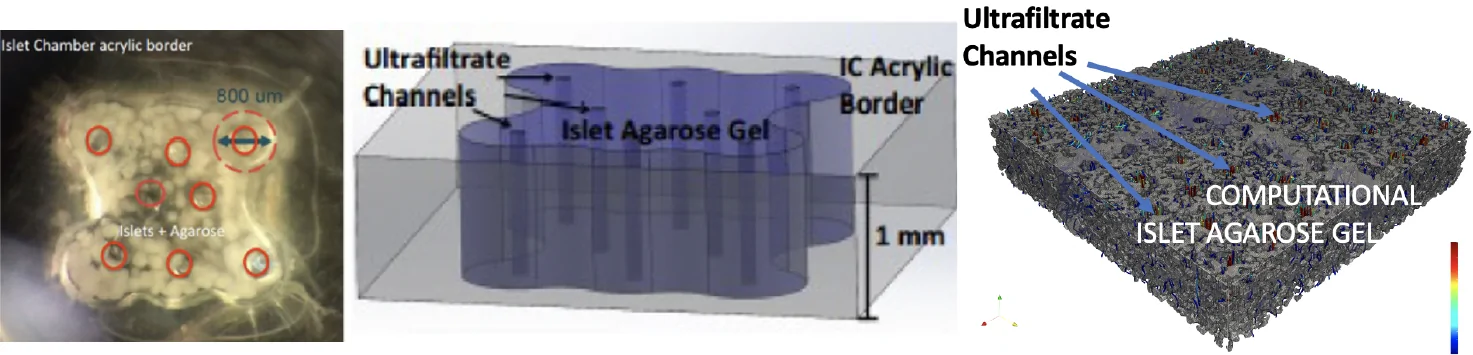
Ultrafiltrate channels in agarose gel.

**Figure 4.**
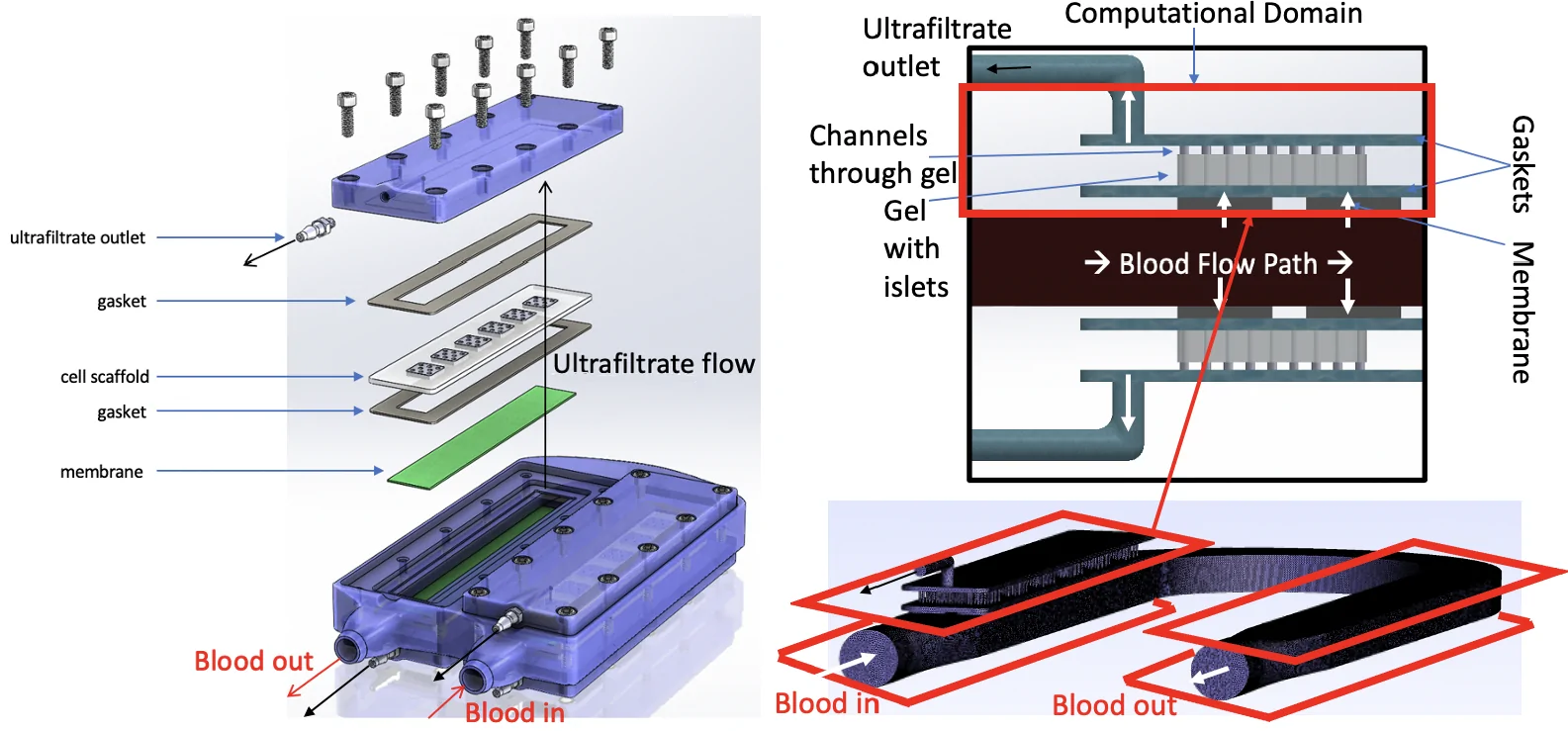
A prototype of a implantable Bioartificial Pancreas Device. The red boxes on the bottom right figure show the location where the four islet chambers are located. Only one of the four chambers is fully depicted in Figure 4 bottom right. A more detailed, magnified sketch of two such chambers is shown on the top figure on the right. The top chamber in the red rectangle shows the computational domain.

**Figure 5.**
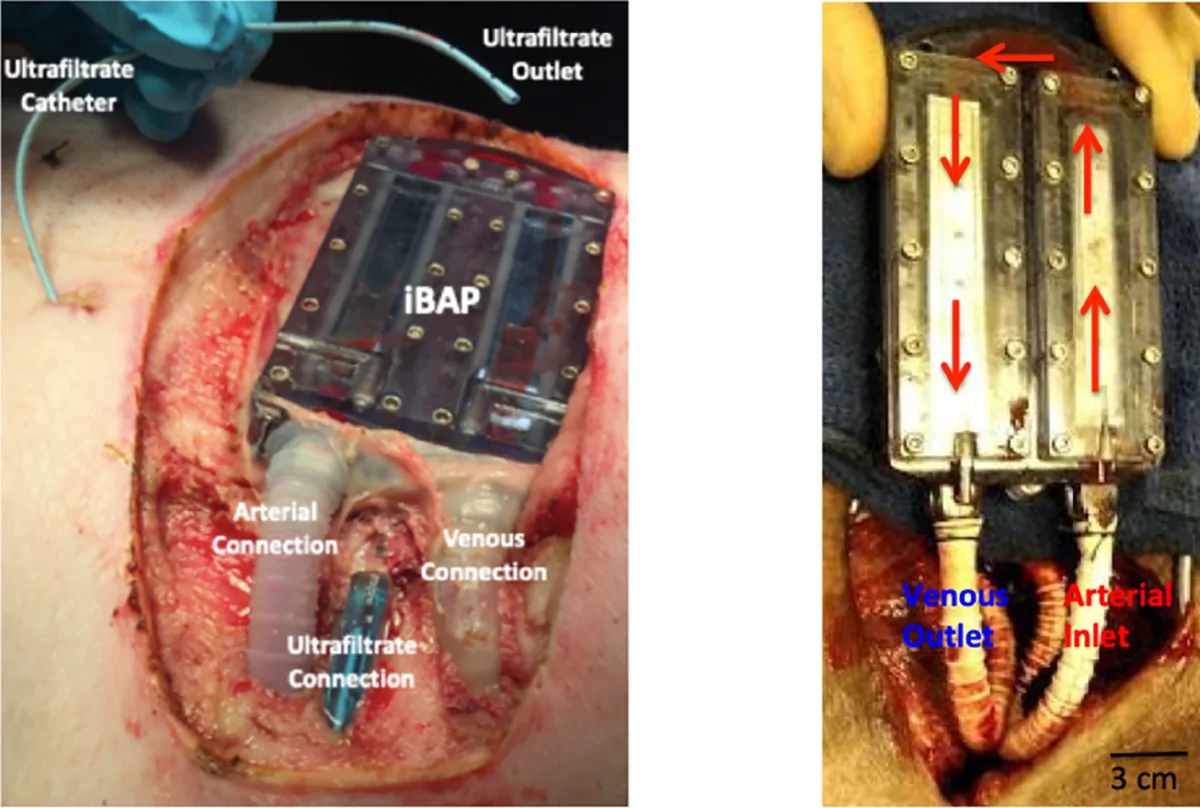
A prototype of an implantable Bioartificial Pancreas (iBAP) implanted into a porcine model.

**Figure 6.**
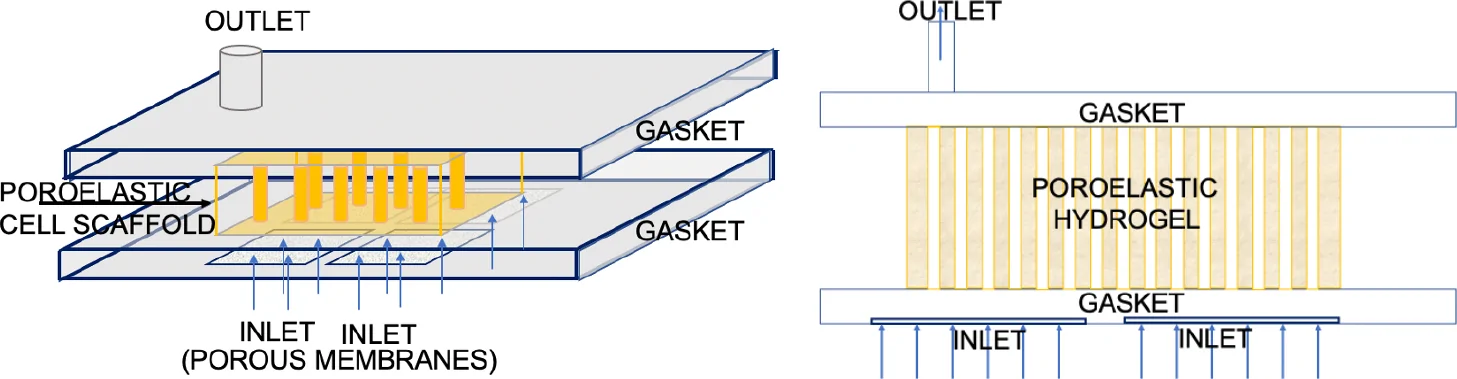
(**Left**): A 3D sketch of the prototype device (computational domain) showing the inlet through four semi-permeable membranes (four blue squares at the bottom), the hydrogel chamber (in orange); ultrafiltrate channels in hydrogel chamber (orange cylinders), and two gaskets—one at the bottom and one at the top of the hydrogel chamber. The anatomosis graft connected to the inlet and outlet is not shown. (**Right**): A 2D slice through the 3D device shown on the left.

**Figure 7.**
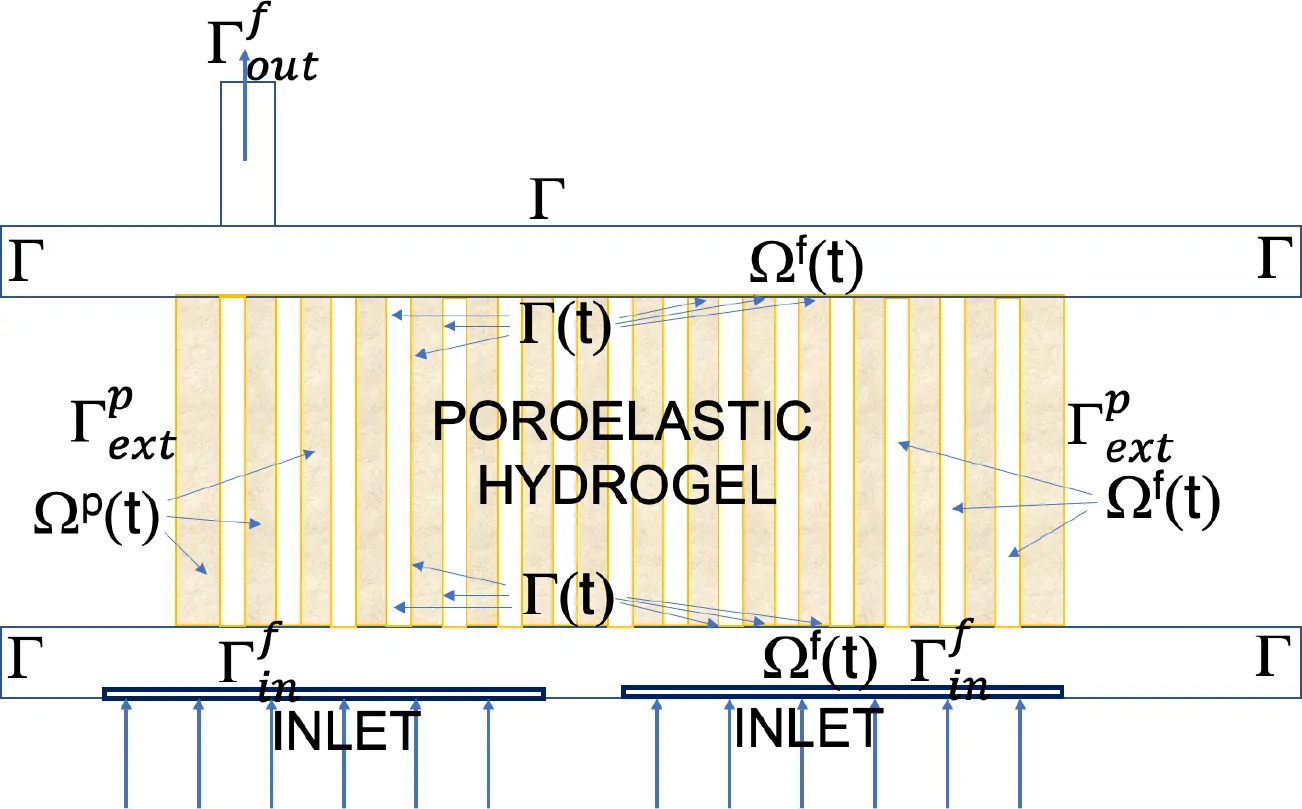
A sketch of a 2D slice of the fluid and poroelastic structure domains together with their boundaries.

**Figure 8.**
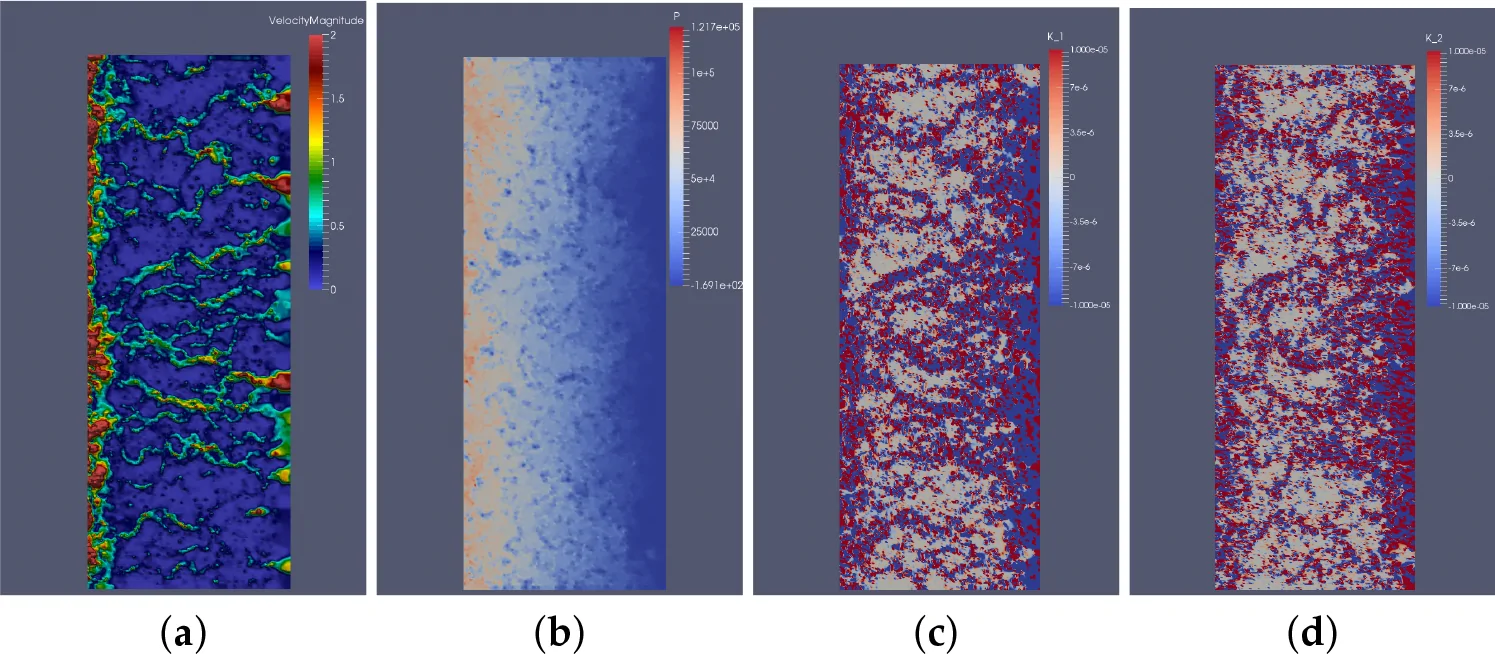
A SPH simulation of a 2D slice of a synthetically generated hydrogel showing: (**a**) Filtration velocity magnitude; (**b**) Pressure field; (**c**) Hydraulic conductivity coefficient in horizontal direction ${\left[\kappa_{0}\right]}_{11}$, and (**d**) in vertical direction ${\left[\kappa_{0}\right]}_{33}$.

**Figure 9.**
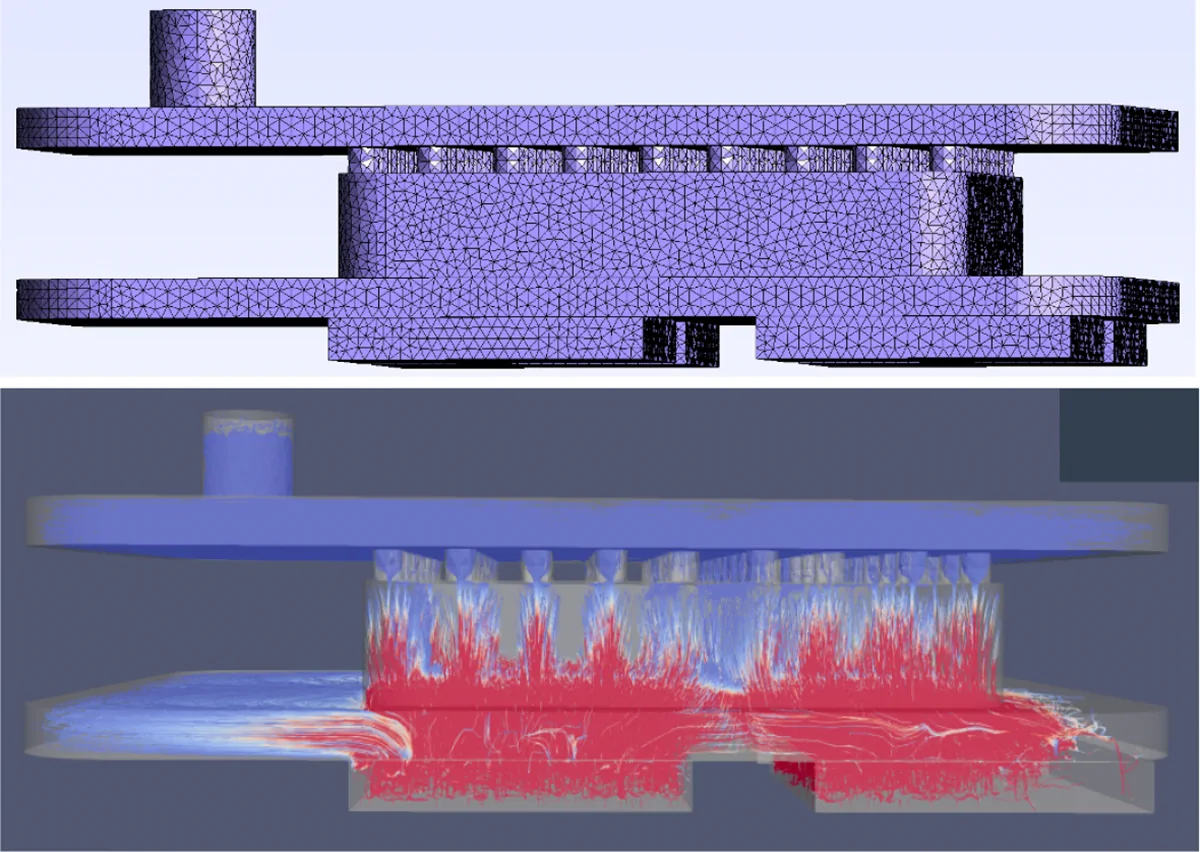
Computational domain and streamlines corresponding to the red box in Figure 4. The inlet is at the bottom through four membranes, and the outlet is at the top through the vertical cylinder. The maximal velocity is 3.5 cm/s (shown in red).

**Figure 10.**
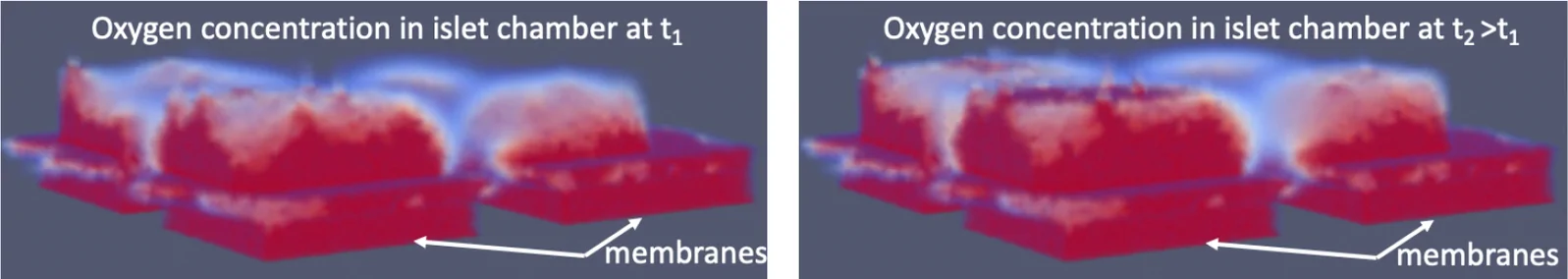
Oxygen concentration at the membranes (inlet) and within the islet chamber for two different times $t_{1}$ and $t_{2}>t_{1}$ as the initial oxygen front convected with the flow enters the bioartificial pancreas. Red denotes high concentration and white and blue low concentration. The flow is from bottom to top. We can see that the regions on the left in both pictures, which are closest to the outlet, get more oxygen than the regions on the right.

**Figure 11.**
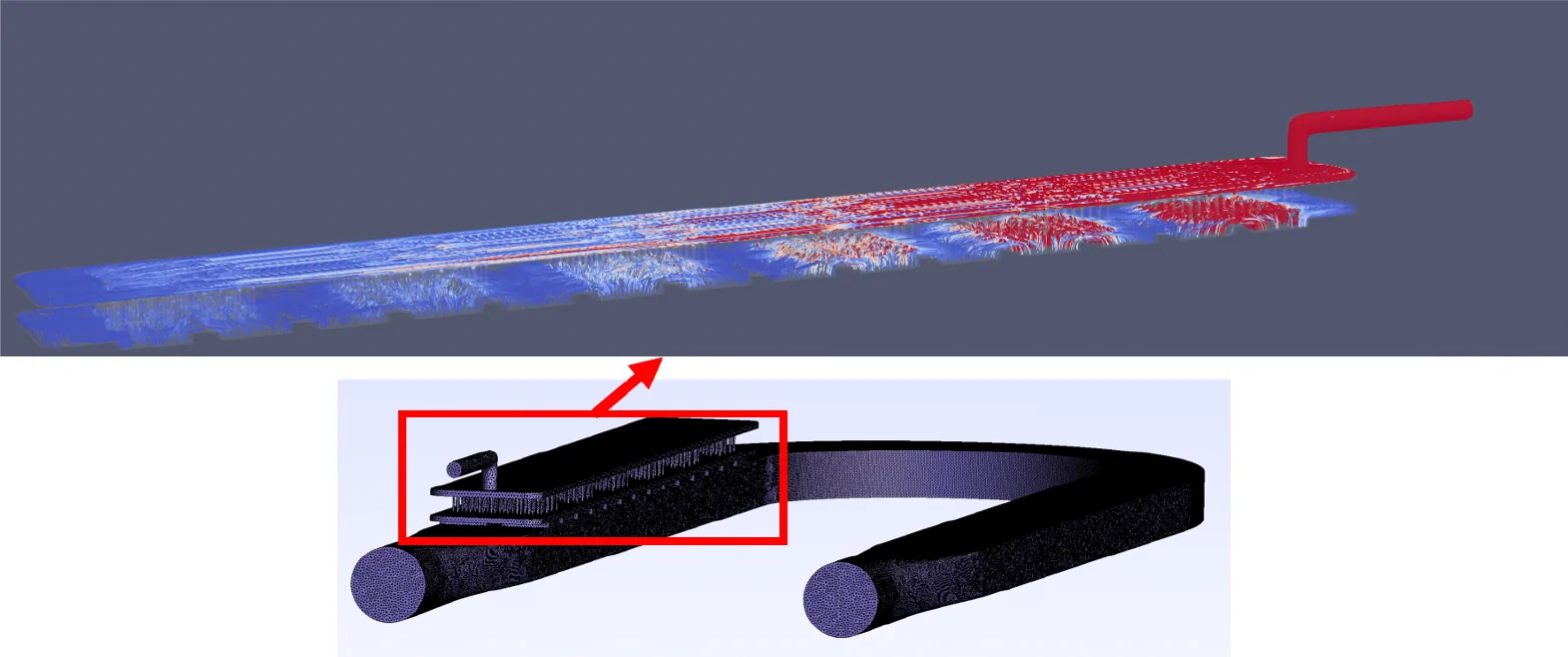
Entire loop. (**Top**): The colors denote the magnitude of fluid velocity with red denoting high velocity (maximum velocity is equal to 3.5 cm/s) and blue denoting low velocity (minimum velocity is 0 cm/s). (**Bottom**): The entire computational domain with mesh.

**Figure 12.**
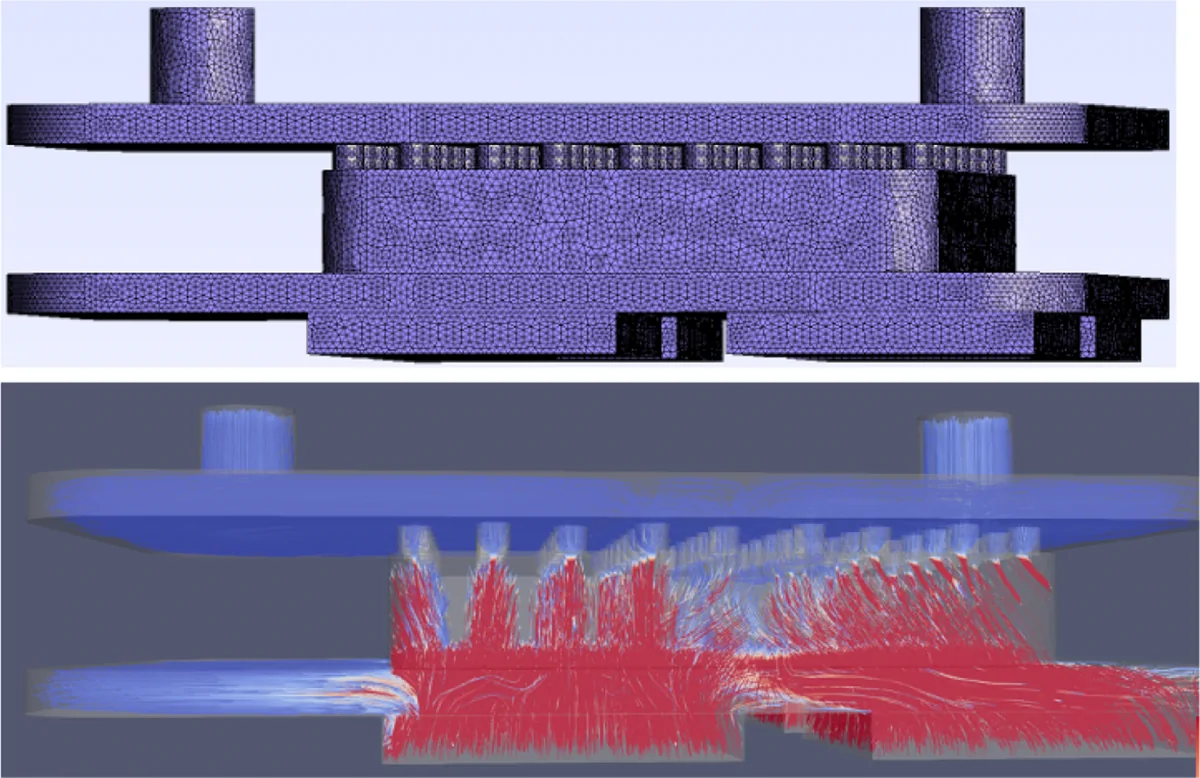
Computational domain and velocity streamlines for the case with two outlets. The maximum velocity is between 3.4 cm/s.

**Figure 13.**
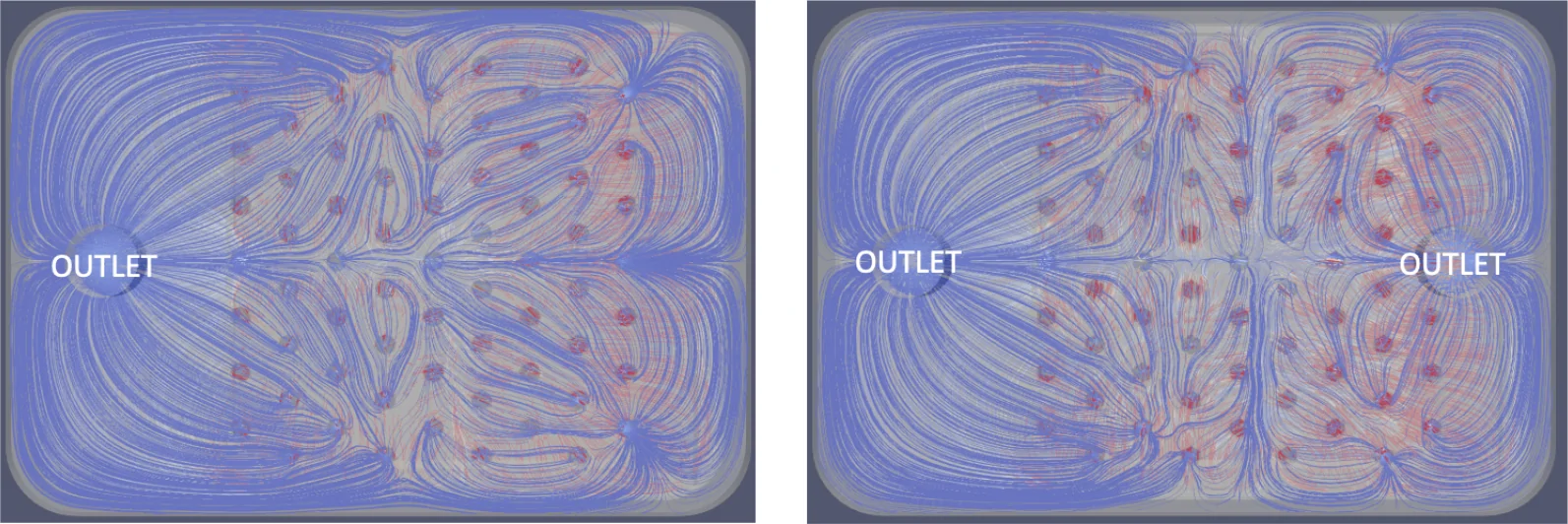
Comparison of the streamlines viewed from the top for the designs with one outlet (**left**), cf. Figure 9, and with two outlets (**right**), cf. Figure 12. The top view shows the outlets, the slow flow (blue) in the top gasket, and the faster flow (red) in the islet chamber. The same velocity scale was used for both pictures. We see stronger flow through the islet chamber on the right (two outlets). In particular, the transverse flow, away from the ultrafiltrate channels, is much stronger in the case of two outlets. This can also be seen in Figure 12 (bottom). A combination of the presence of the second outlet and the “misalignment between the islet chamber and the two inlet membranes in the right half of the chamber is responsible for the increase in transverse (diagonal) flow.

**Figure 14.**
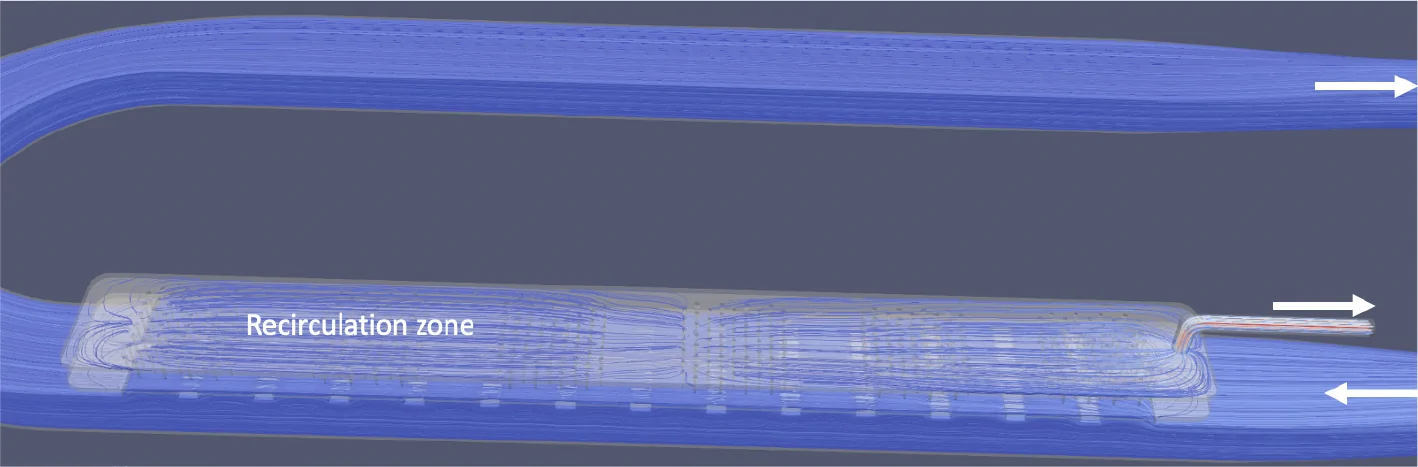
Large recirculation zone in device design with only one outlet.

**Figure 15.**
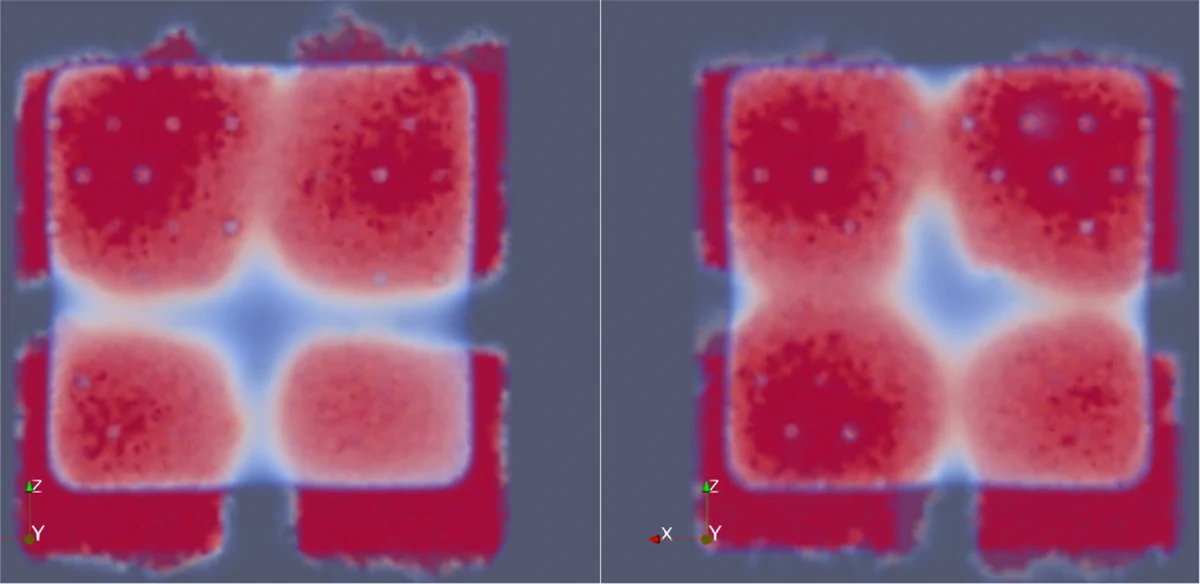
Comparison of oxygen concentration viewed from the top for the designs with one outlet (**left**), cf. Figure 9, and with two outlets (**right**), cf. Figure 12. The same concentration scale was used for both pictures. High oxygen concentration is shown in red, and low in white and blue. We see larger regions of high oxygen concentration in the figure on the right, corresponding to the design with two outlets. The purple dots are the outlines of the ultrafiltrate channels.

**Figure 16.**
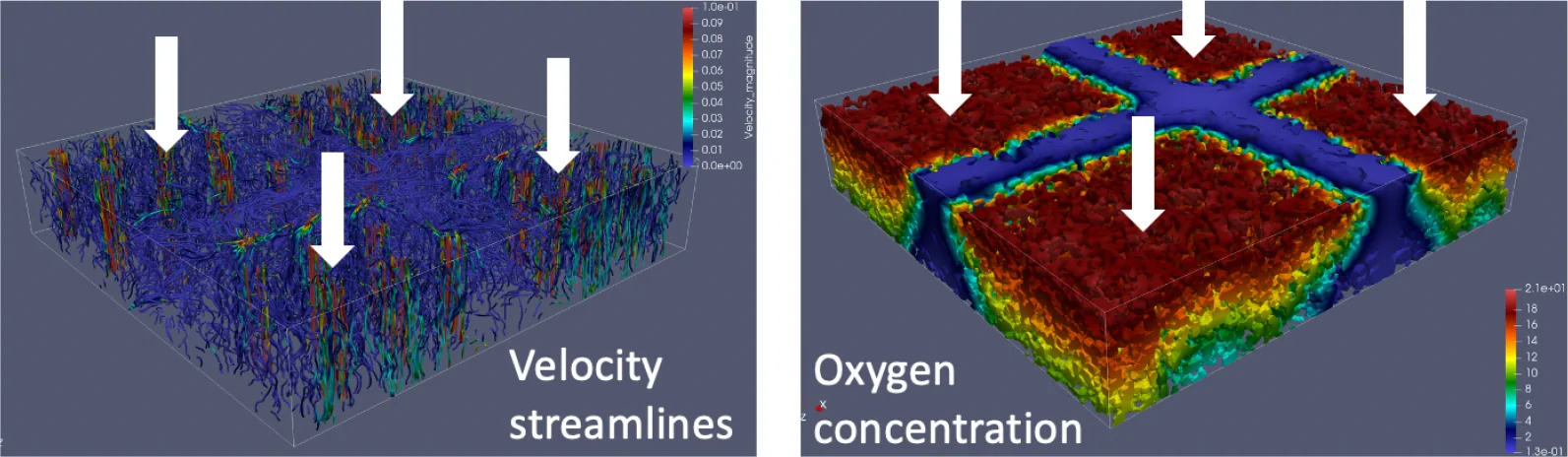
Velocity streamlines colored by velocity magnitude (**left**), and oxygen concentration (**right**) in the poroelastic gel. The direction of flow is from top to bottom. The red streamlines in the figure on the right indicate where the ultrafiltrate channels are located.

**Figure 17.**
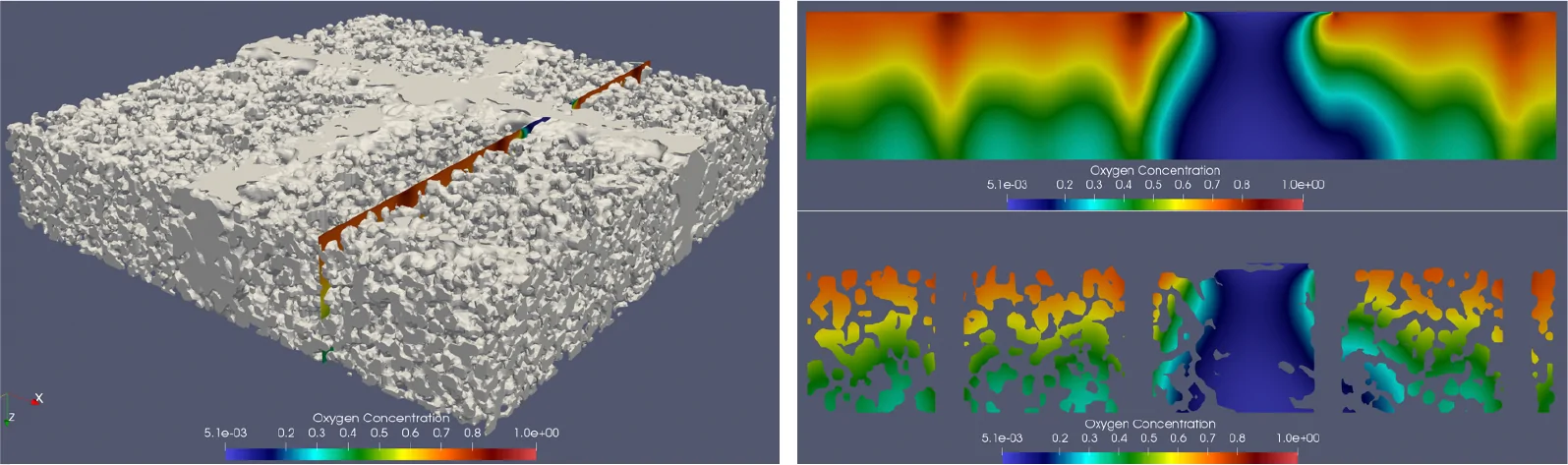
Detailed information about oxygen concentration within the hydrogel for the case with two outlets. (**Left**): 3D hydrogel with a 2D slice. (**Right**): A section of the 2D slice through hydrogel containing details of oxygen concentration (top) and hydrogel structure (bottom). Red is high oxygen concentration and dark blue is low oxygen concentration. Maximum oxygen concentration was 1.06 × 10^−7^ mol/cm^3^. The flow is from top to bottom. The dark red colors with high concentration indicate where the ultrafiltrate channels a located.

**Figure 18.**
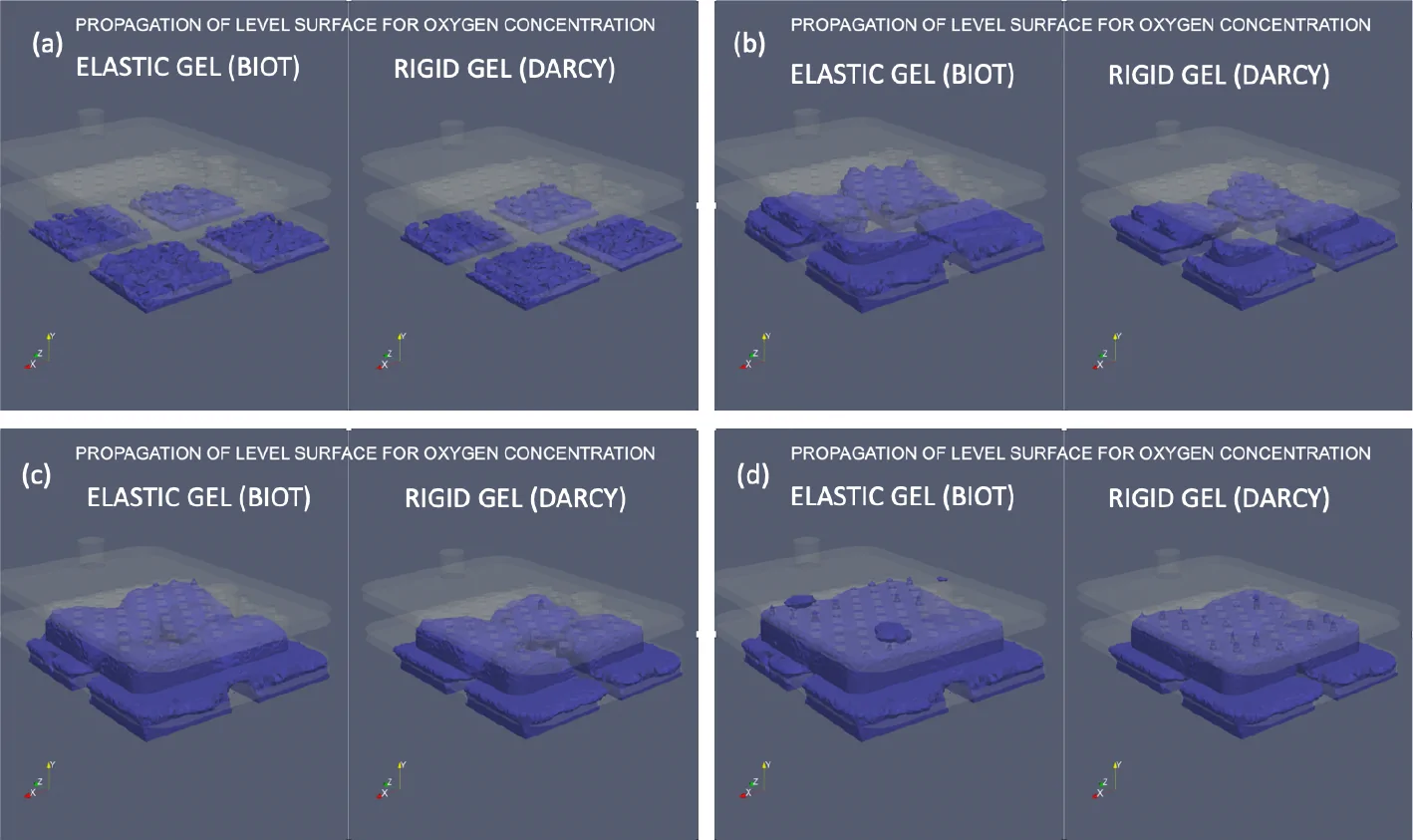
Oxygen front propagation: Comparison between the Biot model (assuming poroelastic hydrogel), and Darcy model (assuming rigid porous hydrogel). The figure shows four snap-shots at increasing times from (**a**–**d**) showing the propagation of a level surface of oxygen concentration. In figure (**d**) we see that oxygen front has already reached the top gasket in the case of the poroelastic Biot model (figure (**d**) left), while in the case of the rigid gel modeled by Darcy law, the front is still within the islet chamber, not yet reaching the top gasket (figure (**d**) right).

**Figure 19.**
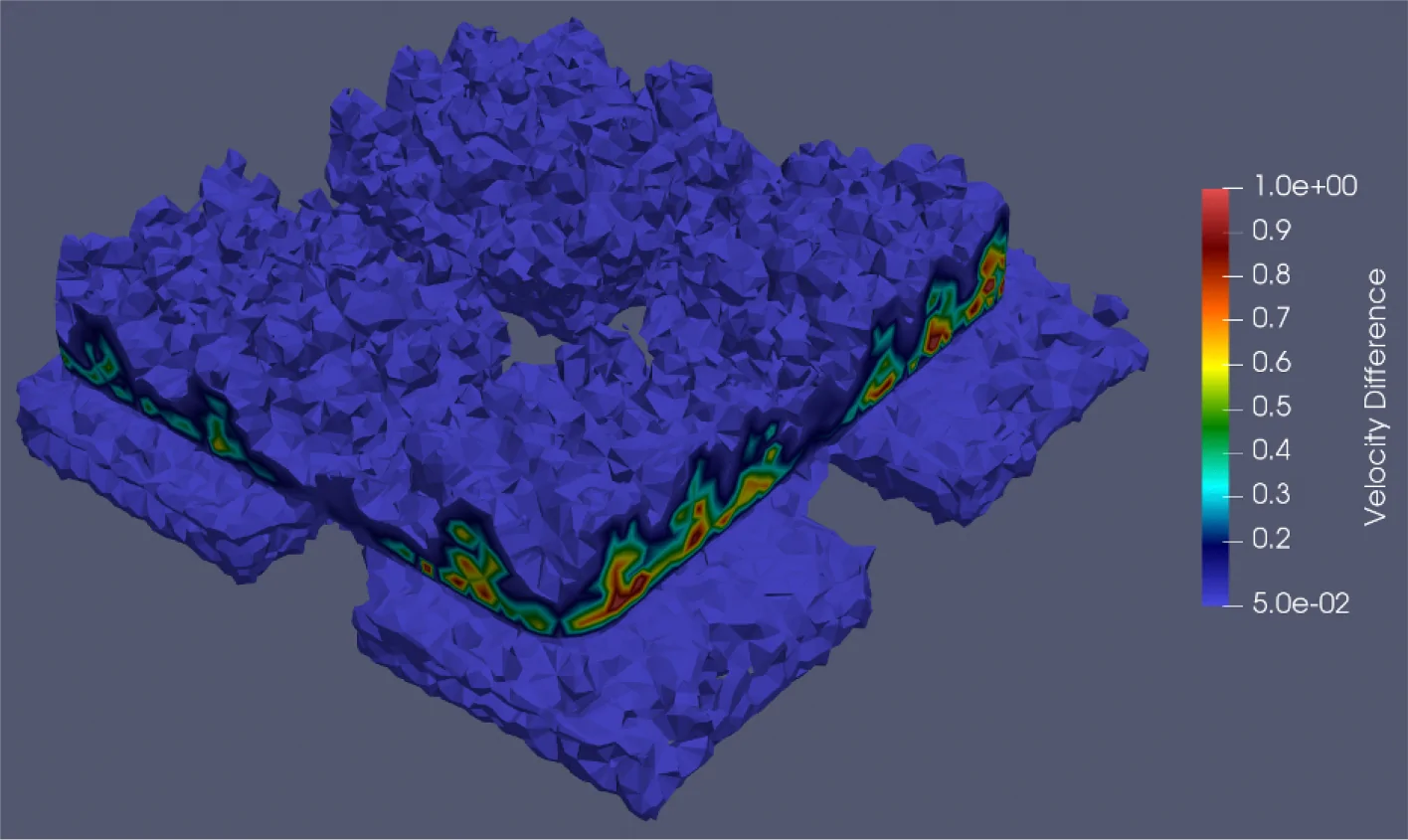
Difference in velocity magnitude between filtration flow through an poroelastic hydrogel, modeled by the Biot model, and rigid hydrogel, modeled by Darcy law. Red is high and blue is low velocity difference. Biot filtration velocity has a higher magnitude, indicating that hydrogel elasticity is conducive to increased filtration flow.

**Figure 20.**
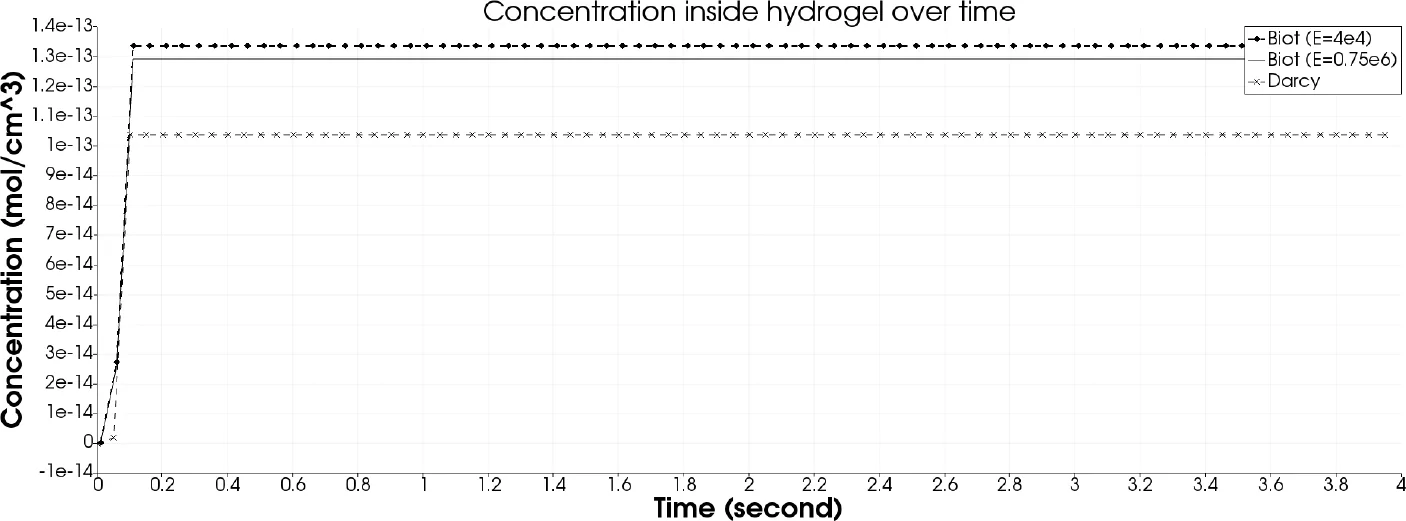
The curves show oxygen concentration over time for three different hydrogels with three different elasticity moduli: alginate hydrogel with Youngs modulus $E=4\times 10^{4}$ Pascals, alginate with $E=0.75\times 10^{6}\;Pa$, and a very stiff scaffold made of Polycaprolactone (PCL) with $E=1.3\times 10^{8}$. We see a significant increase in oxygen concentration for the most elastic alginate hydrogels with $E=4\times 10^{4}\;Pa$ and $E=0.75\times 10^{6}\;Pa$ over the stiff PLC with $E=1.3\times 10^{8}\;Pa$.

**Table 1.** Parameters for FSI simulations.

Parameter	Value

Blood inlet pressure (Average) (mmHg)	46
Blood outlet pressure (Average) (mmHg)	20
Channel height (cm)	0.3
Channel length (cm)	6.5
Channel width (cm)	0.7
Fluid density (g/cm^3^)	1
Fluid viscosity (cm^2^ /s)	0.04
Poroelastic structure density (g/cm^3^)	1.2
Pressure storage coefficient $c_{0}$	1 × 10^−7^
Permeability	2 × 10^−4^
Young’s modulus E (d y n e s / cm^2^)	4 × 10^4^ ~ 0.75 × 10^6^
Poisson’s ratio *σ*	0.49
Biot-Willis parameter *α*	1 × 10^−2^

**Table 2.** Parameters for the coupled convection-reaction-diffusion problem.

Parameters	Value (Units)

Concentration of oxygen at fluid inlet $C_{\mathrm{inlet}}$	2 × 10^−7^ (mol · cm^−3^) [34]
Diffusion coefficient in fluid channel $D_{f}$	3.0 × 10^−5^ (cm^2^ · s^−1^)
Diffusion coefficient in hydrogel $D_{p}$	1.3 × 10^−5^ (cm^2^ · s^−1^)
Maximum oxygen consumption rate $R_{\max}$	3.4 × 10^−8^ (mol · cm^−3^ · s^−1^)
Critical oxygen concentration $C_{\mathrm{cr}}$	1.0 × 10^−10^ (mol · cm^−3^)
The Michaelis–Menten constant $C_{\mathrm{MM}}$	1.0 × 10^−9^ (mol · cm^−3^)

**Table 3.** Temporal convergence test for variables $u_{f}$, $p_{f}$, and $u_{p}$.

Δ*t*	$e_{f}$	Rate	$e_{\mathrm{p,f}}$	Rate	$e_{p}$	Rate

4 × 10^−2^	2.5 × 10^−3^	-	1.2 × 10^−1^	-	0.3127	-
2 × 10^−2^	3.6 × 10^−4^	2.78	2.8 × 10^−2^	2.11	0.0707295	2.14
1 × 10^−2^	9.1 × 10^−5^	1.99	1.4 × 10^−2^	0.96	0.0252249	1.49

**Table 4.** Temporal convergence test for variables $p_{p}$, η and $\dot{\eta }$.

Δ*t*	$e_{\mathrm{p,p}}$	Rate	$e_{\eta }$	Rate	$e_{\dot{\eta }}$	Rate

4 × 10^−2^	1.0 × 10^−2^	-	3.1 × 10^−3^	-	2.6 × 10^−3^	-
2 × 10^−2^	2.5 × 10^−3^	2.16	6.9 × 10^−4^	2.16	5.7 × 10^−4^	2.05
1 × 10^−2^	5.9 × 10^−4^	1.92	1.8 × 10^−4^	1.92	1.5 × 10^−4^	2.07

## Data Availability

Data (parameters) used in the simulations are all presented in the manuscript.
